# Supplementary material for: Redox-Triggered Switching of Conformational State in Triple-Decker Lanthanide Phthalocyaninates
Source: Molecules. 2022 Oct 1;27(19):6498. doi: 10.3390/molecules27196498 (PMC9571987; doi:10.3390/molecules27196498)
Supplement: Supplementary file 1 [file molecules-27-06498-s001.zip › molecules-1921804-supplementary.pdf]

Redox-Triggered Switching of Conformational State in Triple-Decker Lanthanide Phthalocyaninates

Alexander G. Martynov [1,*], Marina A. Polovkova [1], Yulia G. Gorbunova [1,2,*], Aslan Yu. Tsivadze [1,2]

[1] A.N. Frumkin Institute of Physical Chemistry and Electrochemistry, Russian Academy of Sciences, Leninsky pr., 31, Bldg. 4, Moscow, 119071, Russia

[2] N.S. Kurnakov Institute of General and Inorganic Chemistry, Russian Academy of Sciences, Leninsky pr., 31, Moscow, 119991, Russia

* Correspondence: martynov@phyche.ac.ru (A.G.M.) , yulia@igic.ras.ru (Y.G.G.)

ELECTRONIC SUPPORTING INFORMATION

Figure S1. MALDI TOF mass-spectrum of $\mathbf{Tb_2[(BuO)_8Pc]_3}$. 3

Figure S2. ^{1}H-NMR spectrum of $\mathbf{Tb_2[(BuO)_8Pc]_3}$ in $CDCl_3$. 3

Figure S3. MALDI TOF mass-spectrum of $\mathbf{Tb_2[(15C5)_4Pc]_3}$ 4

Figure S4. ^{1}H-NMR spectrum of $\mathbf{Tb_2[(15C5)_4Pc]_3}$ in $CDCl_3$. 4

Figure S5. MALDI TOF mass-spectrum of $\mathbf{Y_2[(BuO)_8Pc]_3}$. 5

Figure S6. ^{1}H-NMR spectrum of $\mathbf{Y_2[(BuO)_8Pc]_3}$ in $CDCl_3$. 5

Figure S7. MALDI TOF mass-spectrum of $\mathbf{Y_2[(15C5)_4Pc]_3}$. 6

Figure S8. ^{1}H-NMR spectrum of $\mathbf{Y_2[(15C5)_4Pc]_3}$ in $CDCl_3$. 6

Figure S9. UV-vis-NIR spectra of neutral forms of $\mathbf{Y_2[(BuO)_8Pc]_3}$ and $\mathbf{Y_2[(15C5)_4Pc]_3}$ and their mono- and dicationic forms, obtained by 1e- and 2e-oxidation with $\mathbf{OxSbCl_6}$. 7

Table S1. The calculated UV-vis-NIR absorption spectrum of ***s*-$\mathbf{Y_2[(MeO)_8Pc]_3}$** for the BP86/def2-SVP geometry calculated by the sTDA method at the CAM-B3LYP/6-31G(d) level of theory. 8

Table S2. The calculated UV-vis-NIR absorption spectrum of ***s*-$\mathbf{Y_2[(MeO)_8Pc]_3^{+}}$** for the BP86/def2-SVP geometry calculated by the sTDA method at the CAM-B3LYP/6-31G(d) level of theory. 9

Table S3. The calculated UV-vis-NIR absorption spectrum of ***s*-$\mathbf{Y_2[(MeO)_8Pc]_3^{2+}}$** for the BP86/def2-SVP geometry calculated by the sTDA method at the CAM-B3LYP/6-31G(d) level of theory. 11

Table S4. The calculated UV-vis-NIR absorption spectrum of ***g*-$\mathbf{Y_2[(MeO)_8Pc]_3}$** for the BP86/def2-SVP geometry calculated by the sTDA method at the CAM-B3LYP/6-31G(d) level of theory. 12

Table S5. The calculated UV-vis-NIR absorption spectrum of ***g*-$\mathbf{Y_2[(MeO)_8Pc]_3^{+}}$** for the BP86/def2-SVP geometry calculated by the sTDA method at the CAM-B3LYP/6-31G(d) level of theory. 13

Table S6. The calculated UV-vis-NIR absorption spectrum of ***g*-Y_2[(MeO)$_8$Pc]$_3^{2+}$** for the BP86/def2-SVP geometry calculated by the sTDA method at the CAM-B3LYP/6-31G(d) level of theory. 16

Figure S10. RDG vs. $sign(\lambda_2)\rho$ plots for staggered and gauche conformations of redox-forms of **Y_2[(MeO)$_8$Pc]$_3$**. 18

Table S7. BP86/def2-SVP geometry of ***s*-Y_2[(MeO)$_8$Pc]$_3$**. 19

Table S8. BP86/def2-SVP geometry of ***s*-Y_2[(MeO)$_8$Pc]$_3^{+}$**. 21

Table S9. BP86/def2-SVP geometry of ***s*-Y_2[(MeO)$_8$Pc]$_3^{2+}$**. 23

Table S10. BP86/def2-SVP geometry of ***g*-Y_2[(MeO)$_8$Pc]$_3$**. 25

Table S11. BP86/def2-SVP geometry of ***g*-Y_2[(MeO)$_8$Pc]$_3^{+}$**. 27

Table S12. BP86/def2-SVP geometry of ***g*-Y_2[(MeO)$_8$Pc]$_3^{2+}$**. 29

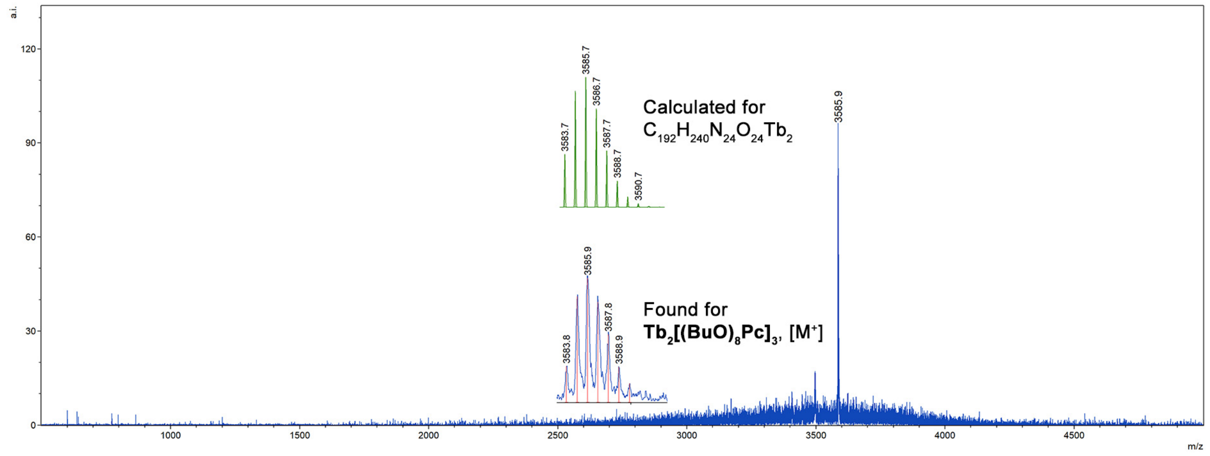

Figure S1. MALDI TOF mass-spectrum of **$Tb_2[(BuO)_8Pc]_3$**.

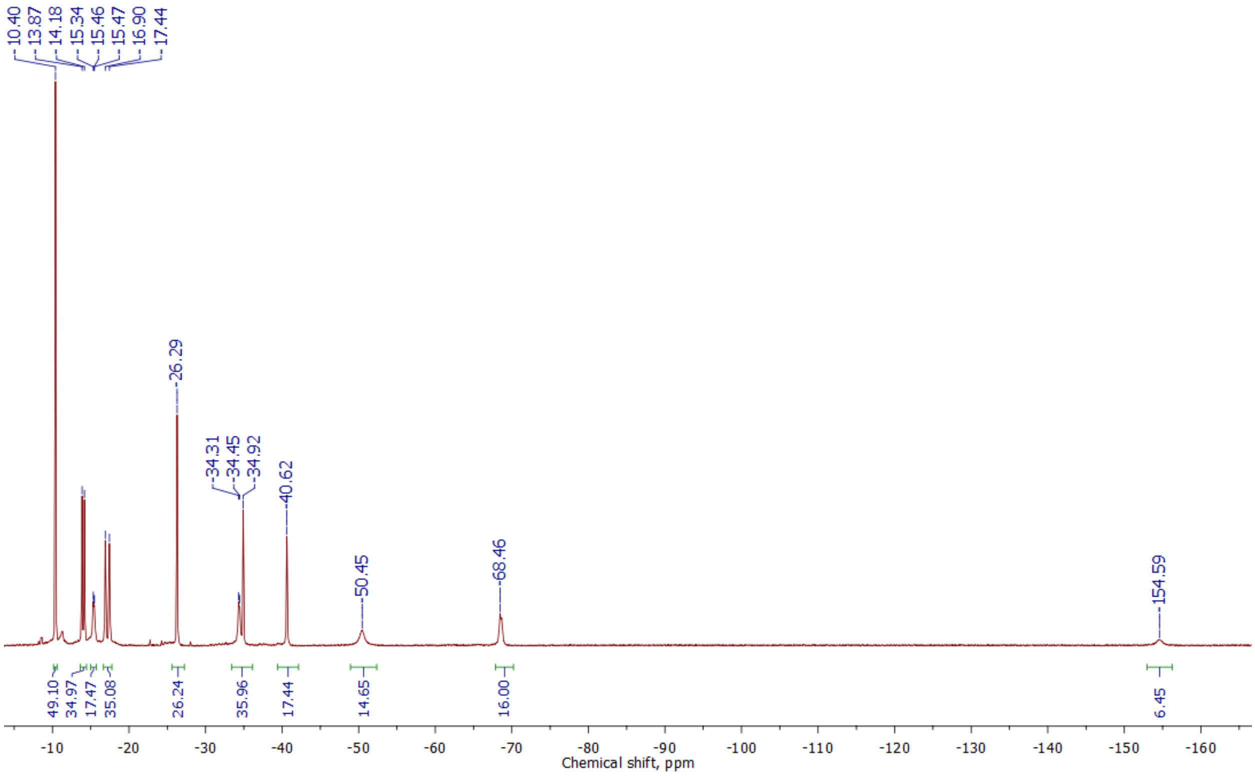

Figure S2. ^{1}H-NMR spectrum of **$Tb_2[(BuO)_8Pc]_3$** in $CDCl_3$.

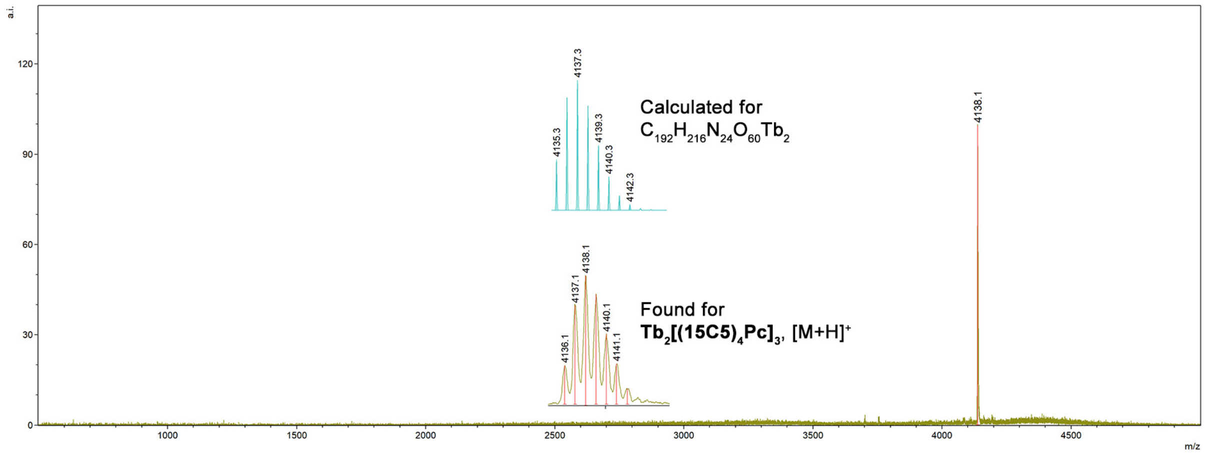

Figure S3. MALDI TOF mass-spectrum of **$Tb_2[(15C5)_4Pc]_3$**

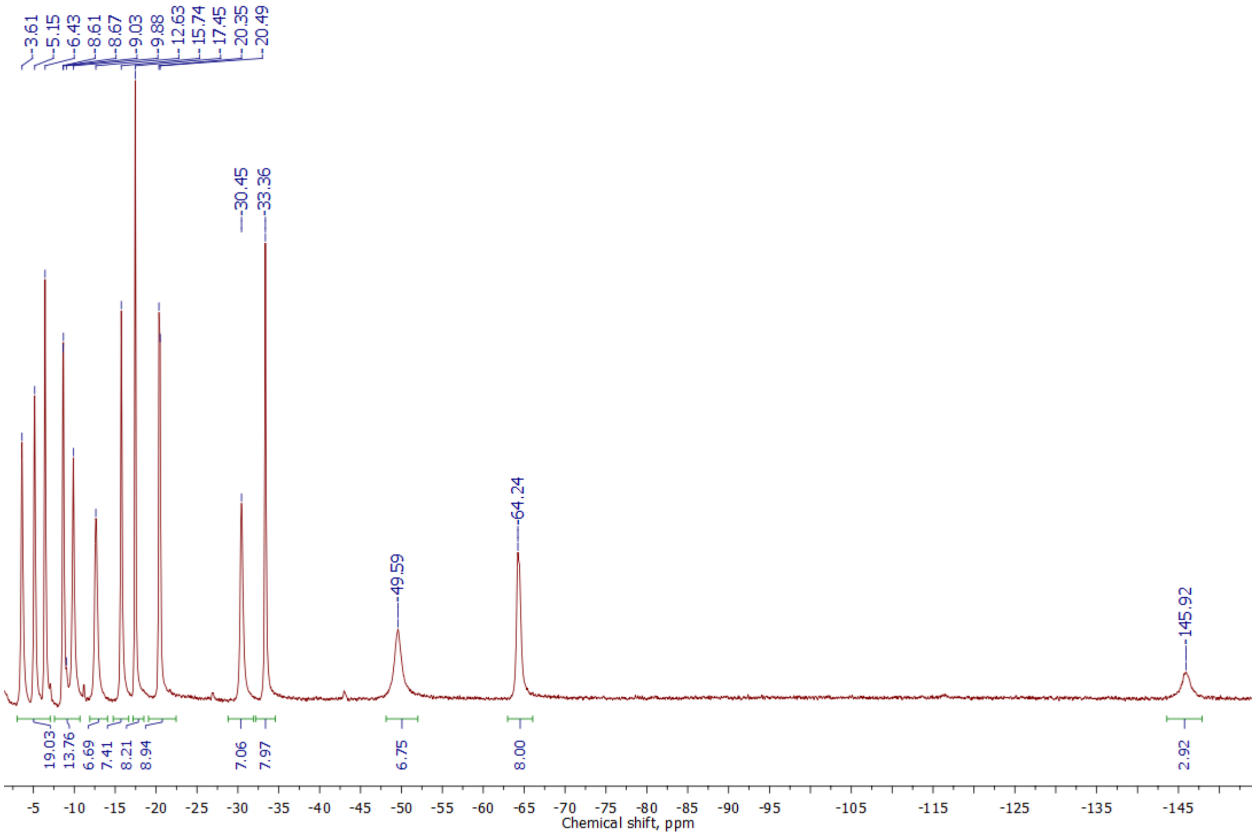

Figure S4. ^{1}H-NMR spectrum of **$Tb_2[(15C5)_4Pc]_3$** in $CDCl_3$.

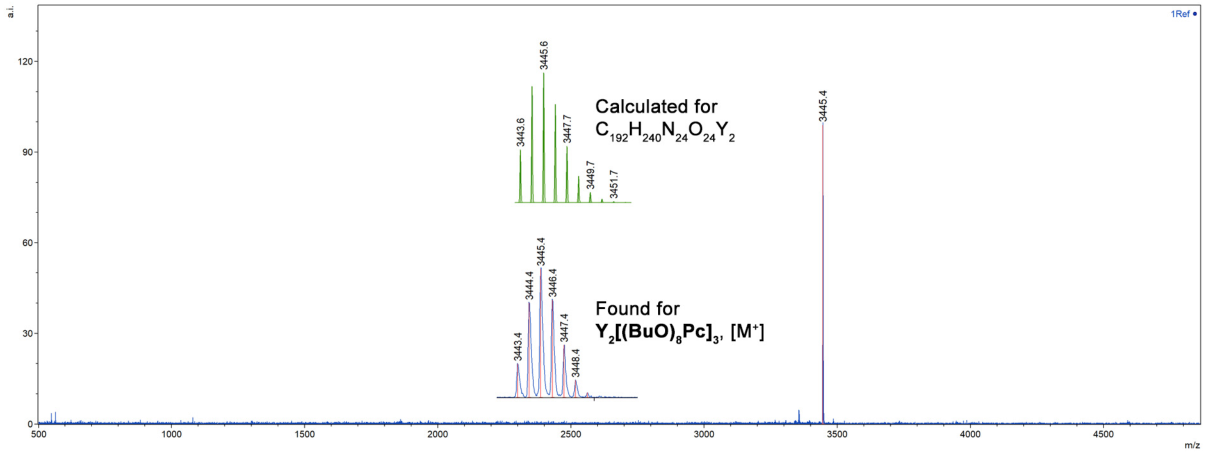

Figure S5. MALDI TOF mass-spectrum of **$Y_2[(BuO)_8Pc]_3$**.

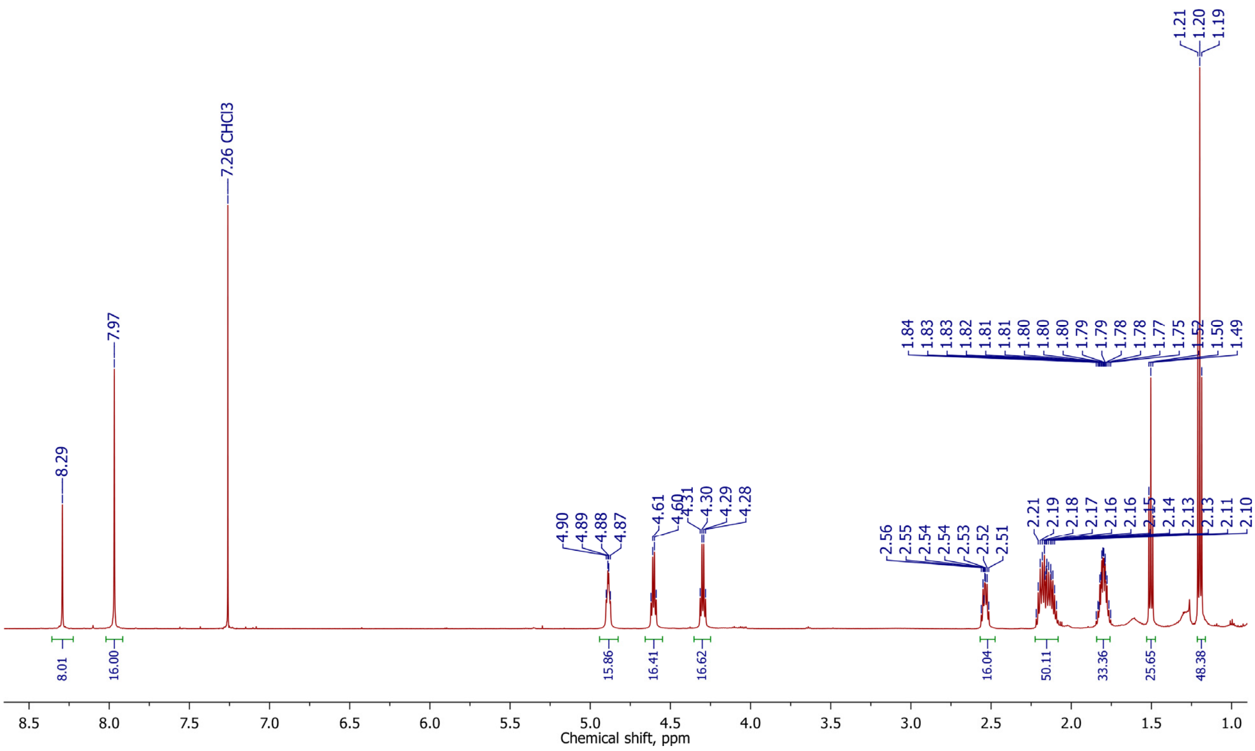

Figure S6. ^{1}H-NMR spectrum of **$Y_2[(BuO)_8Pc]_3$** in $CDCl_3$.

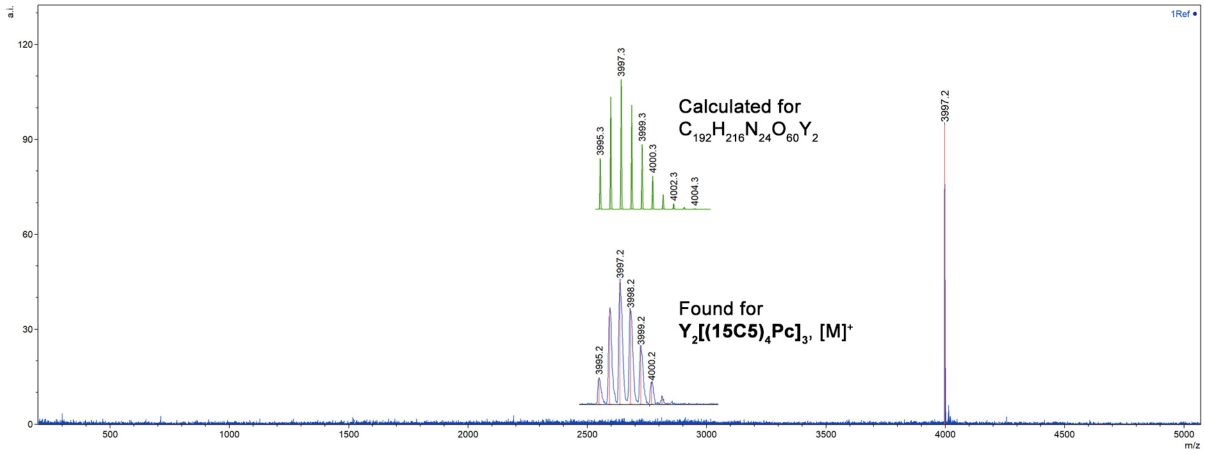

Figure S7. MALDI TOF mass-spectrum of **$Y_2[(15C5)_4Pc]_3$**.

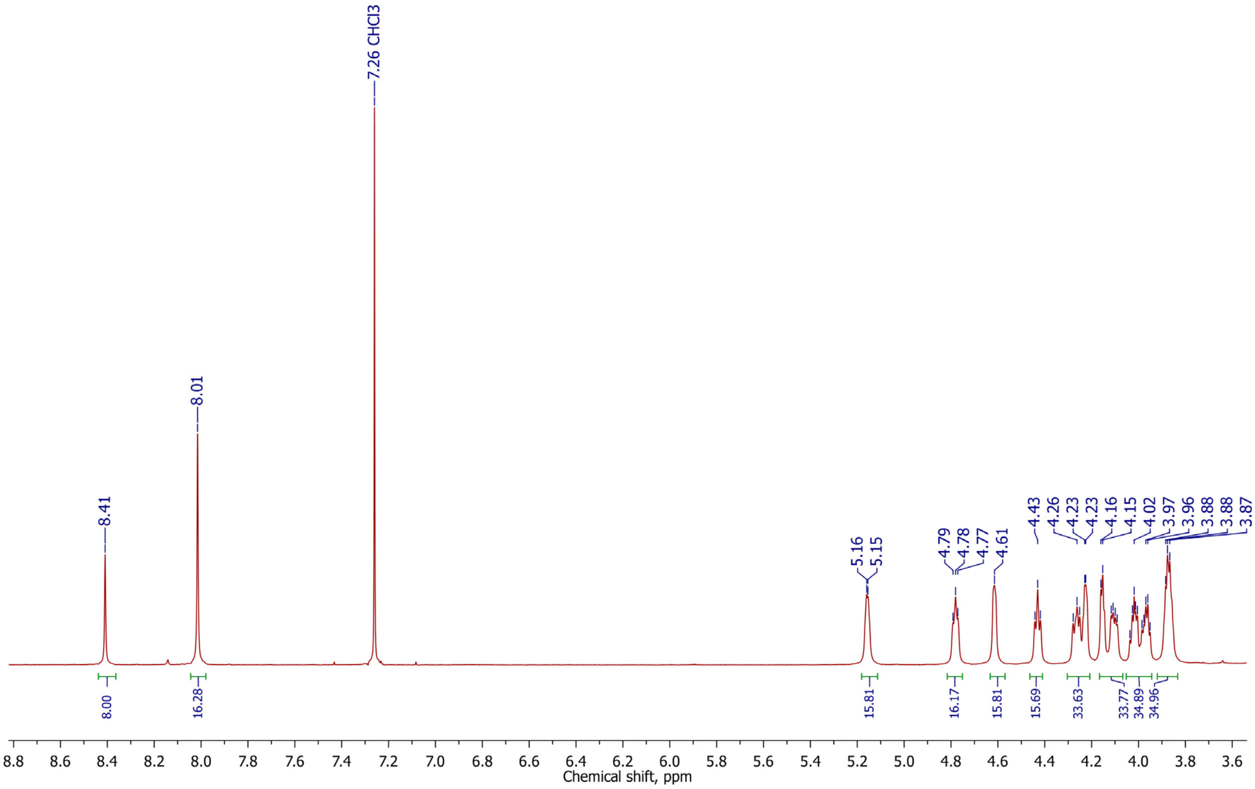

Figure S8. ^{1}H-NMR spectrum of **$Y_2[(15C5)_4Pc]_3$** in $CDCl_3$.

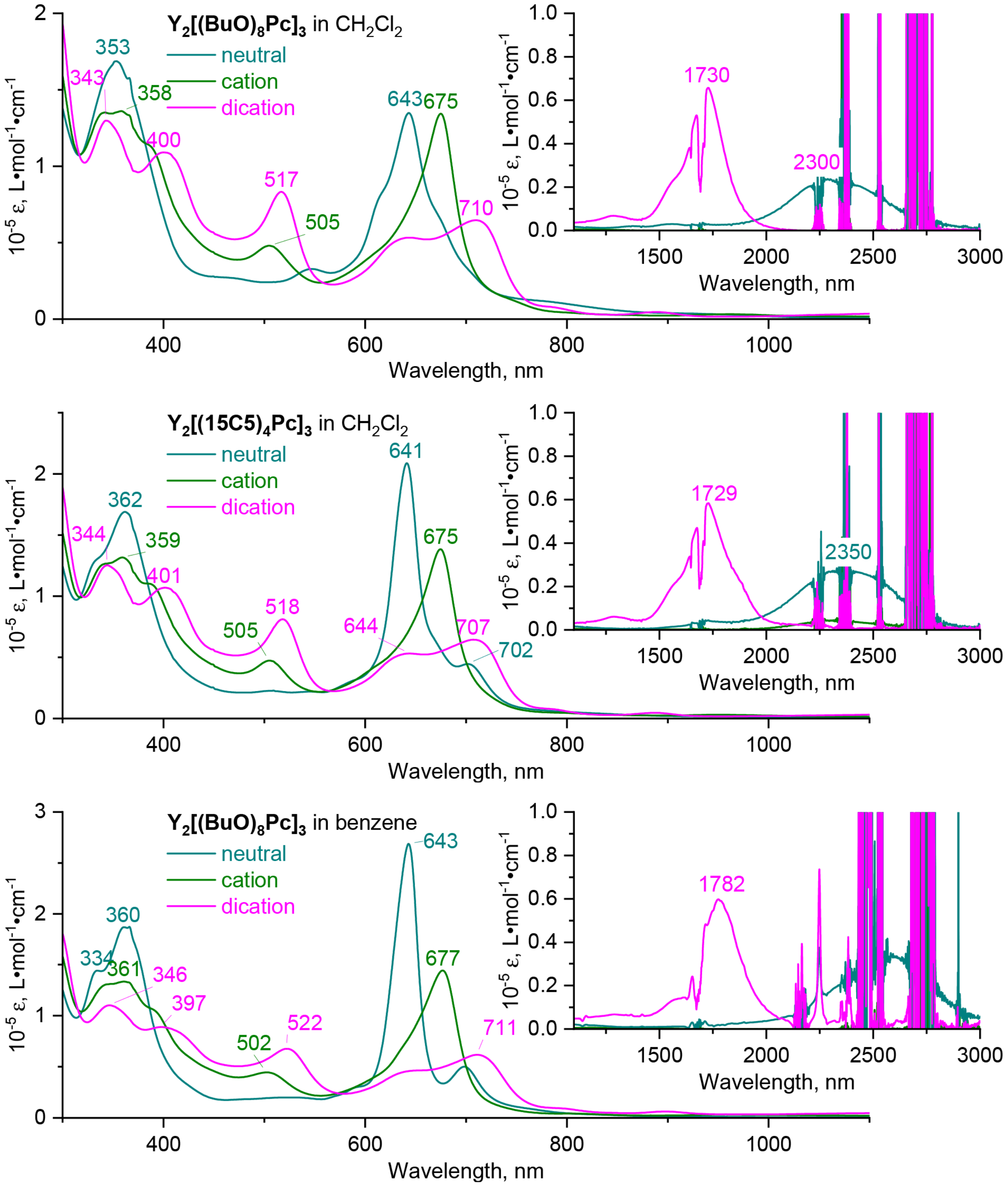

Figure S9. UV-vis-NIR spectra of neutral forms of **$Y_2[(BuO)_8Pc]_3$** and **$Y_2[(15C5)_4Pc]_3$** and their mono- and dicationic forms, obtained by 1e- and 2e-oxidation with **$OxSbCl_6$**.

Table S1. The calculated UV-vis-NIR absorption spectrum of ***s*-Y_2[(MeO)$_8$Pc]$_3$** for the BP86/def2-SVP geometry calculated by the sTDA method at the CAM-B3LYP/6-31G(d) level of theory.

**Wavelength, nm**	**Energy, eV**	**$F_{osc.}$ (1)**	**Wavefunction (2,3)**
932.4	1.33	0.06	-0.83 H→L+5; 0.36 H-2→L; 0.32 H-2→L+1; …
932.4	1.33	0.06	0.83 H→L+4; -0.36 H-2→L+1; 0.32 H-2→L; …
654.0	1.90	0.01	-0.67 H-1→L+2; -0.43 H-1→L+3; 0.4 H-2→L; …
654.0	1.90	0.01	-0.67 H-1→L+3; 0.43 H-1→L+2; -0.4 H-2→L+1; …
579.5	2.14	1.35	0.49 H-2→L+1; -0.39 H-1→L+3; -0.37 H-1→L+2; …
579.5	2.14	1.35	0.49 H-2→L; 0.39 H-1→L+2; -0.37 H-1→L+3; …
527.0	2.35	0.12	0.9 H-3→L; 0.25 H-7→L; -0.21 H-17→L; …
526.9	2.35	0.12	0.9 H-3→L+1; 0.25 H-7→L+1; -0.21 H-17→L+1; …
473.3	2.62	0.03	-0.77 H-8→L; -0.4 H-7→L; -0.35 H-8→L+1; …
473.3	2.62	0.03	-0.77 H-8→L+1; 0.4 H-7→L+1; 0.35 H-8→L; …
457.3	2.71	0.01	0.55 H-11→L+1; 0.55 H-10→L; 0.42 H-10→L+1; …
447.9	2.77	0.06	0.93 H-12→L; 0.26 H-7→L; -0.17 H-7→L+1; …
447.9	2.77	0.06	0.93 H-12→L+1; -0.26 H-7→L+1; -0.17 H-7→L; …
427.3	2.90	0.01	-0.96 H-2→L+5; -0.12 H-3→L+1; 0.08 H-27→L+1; …
427.3	2.90	0.01	0.96 H-2→L+4; -0.12 H-3→L; 0.08 H-27→L; …

(1) Excitations with oscillator strength, $F_{osc.}$ above 0.01 are shown.

(2) Only contributions of greater than 10% are consistently included.

(3) H stands for HOMO, L stands for LUMO

Table S2. The calculated UV-vis-NIR absorption spectrum of ***s*-Y_2[(MeO)$_8$Pc]$_3^+$** for the BP86/def2-SVP geometry calculated by the sTDA method at the CAM-B3LYP/6-31G(d) level of theory.

**Wavelength, nm**	**Energy, eV**	**F$_{osc.}$ (1)**	**Wavefunction (2)**
2491.8	0.50	0.22	-1 bH→bL; -0.02 aH-17→bL+6; -0.02 aH-18→bL+5; …
842.5	1.47	0.03	-0.65 bH-2→bL; 0.52 bH-1→bL+1; 0.48 bH→bL+5; …
842.5	1.47	0.03	0.65 bH-2→bL+1; -0.52 bH-1→bL+2; -0.48 bH→bL+4; …
795.2	1.56	0.03	-0.49 bH-1→bL+1; 0.49 bH→bL+3; 0.48 bH→bL+4; …
795.2	1.56	0.03	0.49 bH-1→bL+2; 0.49 bH→bL+4; -0.48 bH→bL+3; …
662.7	1.87	0.01	-0.59 bH-1→bL+2; 0.48 bH-2→bL+1; 0.39 bH-3→bL; …
662.7	1.87	0.01	0.59 bH-1→bL+3; -0.48 bH-2→bL; 0.39 bH-3→bL+1; …
623.9	1.99	0.66	-0.48 bH-1→bL+3; -0.36 aH-12→bL; -0.35 bH-2→bL; …
623.9	1.99	0.66	-0.48 bH-1→bL+2; 0.36 aH-13→bL; -0.35 bH-2→bL+1; …
596.0	2.08	0.10	0.85 bH-5→bL; 0.19 bH-3→bL; 0.17 bH-3→bL+1; …
596.0	2.08	0.10	-0.85 bH-4→bL; -0.19 bH-3→bL+1; 0.17 bH-3→bL; …
564.7	2.20	0.03	0.65 aH-9→bL; 0.48 bH-3→bL; -0.26 aH-8→bL+1; …
564.7	2.20	0.03	0.65 aH-9→bL+1; 0.48 bH-3→bL+1; -0.26 aH-8→bL; …
543.3	2.28	0.21	-0.57 bH-2→bL+1; 0.44 aH-9→bL; 0.33 aH-11→bL+1; …
543.3	2.28	0.21	0.57 bH-2→bL+2; -0.44 aH-9→bL+1; -0.33 aH-11→bL+2; …
535.5	2.32	0.01	0.53 aH-11→bL; -0.52 aH-10→bL+1; -0.43 aH-11→bL+1; …
526.4	2.36	0.15	-0.46 bH-2→bL+2; -0.44 aH-12→bL; -0.36 aH-13→bL; …
526.4	2.36	0.15	-0.46 bH-2→bL+1; -0.44 aH-13→bL; 0.36 aH-12→bL; …
515.1	2.41	0.01	0.41 bH-7→bL+1; -0.41 aH-11→bL+2; 0.34 aH-14→bL+1; …
515.1	2.41	0.01	0.41 aH-11→bL+1; -0.41 bH-7→bL+2; 0.34 aH-14→bL; …
508.4	2.44	0.03	0.52 aH-14→bL+1; 0.43 aH-14→bL; -0.42 bH-7→bL+1; …
508.4	2.44	0.03	0.52 aH-14→bL; -0.43 aH-14→bL+1; 0.42 bH-7→bL+2; …
503.1	2.46	0.02	-0.47 aH-11→bL+1; -0.45 bH-7→bL+2; -0.38 aH-11→bL+2; …
503.1	2.47	0.02	0.47 aH-11→bL+2; 0.45 bH-7→bL+1; -0.38 aH-11→bL+1; …
491.7	2.52	0.01	-0.51 aH-10→bL+1; 0.51 aH-9→bL+2; 0.44 aH-10→bL+2; …
461.2	2.69	0.09	-0.9 aH-14→bL+1; 0.3 aH-14→bL; -0.19 aH-13→bL; …
461.2	2.69	0.09	0.9 aH-14→bL+2; -0.3 aH-14→bL+1; -0.19 aH-12→bL; …
427.7	2.90	0.01	0.51 aH-16→bL; 0.49 aH-19→bL+1; -0.28 aH-22→bL+2; …
427.7	2.90	0.01	-0.51 aH-15→bL; 0.49 aH-19→bL; -0.28 aH-22→bL+1; …
422.6	2.93	0.02	-0.55 bH-4→bL+2; -0.31 bH-4→bL+3; 0.3 aH-8→bL+4; …
422.6	2.93	0.02	0.55 bH-4→bL+3; -0.31 bH-4→bL+2; -0.3 aH-8→bL+5; …
417.2	2.97	0.26	0.57 aH-16→bL; -0.5 aH-19→bL+1; 0.34 aH-15→bL; …
417.2	2.97	0.26	-0.57 aH-15→bL; -0.5 aH-19→bL; 0.34 aH-16→bL; …
409.2	3.03	0.04	-0.58 bH-2→bL+4; -0.52 bH-1→bL+5; 0.28 bH-3→bL+5; …

409.2	3.03	0.04	0.58 bH-2→bL+5; 0.52 bH-1→bL+6; 0.28 bH-3→bL+4; …
402.4	3.08	0.13	-0.33 bH-4→bL+3; -0.33 bH-7→bL+3; -0.32 bH-3→bL+5; …
402.4	3.08	0.13	0.33 bH-4→bL+2; -0.32 bH-7→bL+2; -0.32 bH-3→bL+4; …
401.2	3.09	0.01	-0.52 bH-5→bL+2; -0.52 bH-6→bL+3; -0.24 bH-5→bL+3; …

(1) Excitations with oscillator strength, $F_{osc.}$ above 0.01 are shown.

(2) H stands for HOMO, L stands for LUMO

Table S3. The calculated UV-vis-NIR absorption spectrum of **s-Y_2[(MeO)$_8$Pc]$_3^{2+}$** for the BP86/def2-SVP geometry calculated by the sTDA method at the CAM-B3LYP/6-31G(d) level of theory.

**Wavelength, nm**	**Energy, eV**	**$F_{osc.}$ (1)**	**Wavefunction (2)**
1616.0	0.77	0.74	-0.99 H→L; 0.03 H-8→L+2; -0.03 H-9→L+1
748.1	1.66	0.01	-0.56 H-1→L+1; -0.52 H→L+3; -0.48 H→L+4
748.1	1.66	0.01	-0.56 H-1→L+2; -0.52 H→L+4; 0.48 H→L+3
701.8	1.77	0.42	0.58 H→L+4; -0.57 H-1→L+2; -0.33 H-12→L
701.8	1.77	0.42	0.58 H→L+3; -0.57 H-1→L+1; -0.33 H-11→L
691.4	1.79	0.01	-0.94 H-6→L; 0.17 H-12→L; -0.11 H→L+3
603.9	2.05	0.03	-0.87 H-2→L+1; 0.37 H-10→L+1; 0.2 H-1→L+2
603.9	2.05	0.03	0.87 H-2→L+2; -0.37 H-10→L+2; 0.2 H-1→L+1
579.7	2.14	0.02	-0.67 H-7→L+1; -0.38 H-11→L; -0.37 H-10→L+1
579.7	2.14	0.02	-0.67 H-7→L+2; 0.38 H-12→L; 0.37 H-10→L+2
570.2	2.17	0.42	-0.49 H-10→L+2; -0.47 H-10→L+1; -0.33 H-7→L+2
570.2	2.17	0.42	-0.49 H-10→L+1; 0.47 H-10→L+2; 0.33 H-7→L+1
568.3	2.18	0.21	-0.64 H-11→L; 0.53 H-7→L+1; 0.25 H-10→L+2
568.3	2.18	0.21	0.64 H-12→L; 0.53 H-7→L+2; 0.25 H-10→L+1
558.5	2.22	0.02	-0.58 H-9→L+1; 0.58 H-8→L+2; 0.39 H-9→L+2
501.5	2.47	0.10	0.95 H-13→L+2; -0.15 H-11→L; -0.13 H-13→L+1
501.5	2.47	0.10	0.95 H-13→L+1; -0.15 H-12→L; 0.13 H-13→L+2
445.5	2.78	0.45	0.8 H-15→L; -0.47 H-14→L; 0.12 H-21→L+2
445.5	2.78	0.45	-0.8 H-14→L; -0.47 H-15→L; -0.12 H-21→L+1
404.8	3.06	0.01	0.76 H-4→L+3; 0.29 H-3→L+4; -0.27 H-7→L+6
404.8	3.06	0.01	-0.76 H-4→L+4; -0.29 H-3→L+3; 0.27 H-7→L+5

(1) Excitations with oscillator strength, $F_{osc.}$ above 0.01 are shown.

(2) H stands for HOMO, L stands for LUMO

Table S4. The calculated UV-vis-NIR absorption spectrum of ***g*-Y_2[(MeO)$_8$Pc]$_3$** for the BP86/def2-SVP geometry calculated by the sTDA method at the CAM-B3LYP/6-31G(d) level of theory.

Wavelength, nm	Energy, eV	$F_{osc.}$ (1)	Wavefunction (2)
1613.3	0.77	0.02	-0.97 H→L; -0.18 H→L+5; 0.06 H→L+4
1613.2	0.77	0.02	-0.97 H→L+1; -0.18 H→L+4; -0.06 H→L+5
1052.5	1.18	0.03	0.87 H-2→L; -0.37 H-1→L; 0.15 H-1→L+5
1052.5	1.18	0.03	0.87 H-2→L+1; -0.37 H-1→L+1; 0.15 H-1→L+4
807.6	1.54	0.01	0.66 H-2→L+3; -0.63 H-1→L+3; 0.29 H→L+2
807.6	1.54	0.01	-0.66 H-2→L+2; 0.63 H-1→L+2; 0.29 H→L+3
709.2	1.75	0.03	-0.66 H→L+2; -0.61 H→L+3; -0.22 H-1→L+3
709.2	1.75	0.03	-0.66 H→L+3; 0.61 H→L+2; 0.22 H-1→L+2
686.8	1.81	0.10	-0.53 H→L+5; 0.49 H-2→L+4; -0.46 H-1→L+4
686.8	1.81	0.10	0.53 H→L+4; 0.49 H-2→L+5; -0.46 H-1→L+5
577.5	2.15	0.24	0.74 H-1→L+5; -0.3 H-1→L+4; 0.27 H-2→L+5
577.5	2.15	0.24	-0.74 H-1→L+4; -0.3 H-1→L+5; -0.27 H-2→L+4
550.4	2.25	0.82	0.67 H-2→L+4; 0.3 H→L+5; 0.28 H-2→L+5
550.4	2.25	0.82	-0.67 H-2→L+5; 0.3 H→L+4; 0.28 H-2→L+4
526.2	2.36	0.01	-0.92 H-3→L+1; 0.21 H-6→L+1; -0.19 H-3→L
526.2	2.36	0.01	0.92 H-3→L; 0.21 H-6→L; -0.19 H-3→L+1
496.3	2.50	0.05	0.81 H-6→L+1; -0.33 H-6→L; 0.22 H-3→L
496.2	2.50	0.05	-0.81 H-6→L; -0.33 H-6→L+1; -0.22 H-3→L+1
474.8	2.61	0.02	-0.88 H-7→L+1; -0.32 H-7→L; -0.2 H-3→L+3
474.8	2.61	0.02	0.88 H-7→L; -0.32 H-7→L+1; -0.2 H-3→L+2
440.7	2.81	0.02	-0.56 H-11→L; 0.55 H-15→L; -0.3 H-10→L+1
440.7	2.81	0.02	-0.56 H-11→L+1; 0.55 H-15→L+1; 0.3 H-10→L
436.3	2.84	0.09	-0.81 H-10→L; -0.45 H-11→L+1; -0.17 H-6→L+2
436.3	2.84	0.09	0.81 H-10→L+1; -0.45 H-11→L; -0.17 H-6→L+3
413.9	3.00	0.02	-0.78 H-12→L+1; -0.29 H-11→L+1; -0.23 H-7→L+3
413.9	3.00	0.02	0.78 H-12→L; -0.29 H-11→L; -0.23 H-7→L+2
407.1	3.05	0.01	-0.64 H-13→L+1; -0.64 H-14→L; -0.21 H-13→L

(1) Excitations with oscillator strength, $F_{osc.}$ above 0.01 are shown.

(2) H stands for HOMO, L stands for LUMO

Table S5. The calculated UV-vis-NIR absorption spectrum of ***g*-Y_2[(MeO)$_8$Pc]$_3^+$** for the BP86/def2-SVP geometry calculated by the sTDA method at the CAM-B3LYP/6-31G(d) level of theory.

Wavelength, nm	Energy, eV	$F_{osc.}$ (1)	Wavefunction (2)
1160.6	1.07	0.01	-0.69 bH-1→bL+1; 0.52 bH-1→bL; 0.42 bH-1→bL+2
1093.9	1.13	0.01	0.8 bH-1→bL+2; 0.43 bH-1→bL+3; -0.36 bH-1→bL+1
896.6	1.38	0.02	0.68 bH-2→bL; -0.29 bH→bL+2; 0.29 bH-2→bL
889.5	1.39	0.01	-0.61 bH-2→bL+1; -0.39 bH-1→bL+2; 0.35 bH-4→bL
875.4	1.42	0.01	0.59 bH-4→bL; 0.53 bH-2→bL+1; -0.42 aH-9→bL
828.7	1.50	0.05	-0.66 bH-1→bL+2; -0.36 bH-1→bL+1; -0.34 bH-4→bL
827.6	1.50	0.01	0.76 bH-5→bL; -0.42 aH-10→bL; 0.24 bH-7→bL
815.2	1.52	0.04	-0.76 bH-1→bL+3; 0.38 bH-1→bL+2; 0.28 bH-3→bL
739.4	1.68	0.06	0.63 bH→bL+4; -0.31 bH→bL+3; 0.28 bH→bL+6
738.4	1.68	0.06	0.61 bH→bL+3; 0.33 bH→bL+4; -0.28 bH→bL+5
718.4	1.73	0.04	0.46 bH-7→bL; -0.43 bH-3→bL; 0.33 bH→bL+3
717.9	1.73	0.04	0.47 aH-9→bL; 0.44 bH-4→bL; -0.29 bH→bL+4
680.7	1.82	0.02	-0.87 bH-1→bL+3; 0.19 bH-1→bL+4; -0.16 bH-2→bL+2
673.0	1.84	0.01	-0.74 bH-1→bL+4; -0.3 bH-5→bL; -0.28 aH-11→bL
638.8	1.94	0.18	-0.55 bH→bL+5; 0.47 bH→bL+4; -0.35 bH→bL+6
637.6	1.95	0.17	0.54 bH→bL+6; 0.47 bH→bL+5; -0.36 bH→bL+5
630.2	1.97	0.02	-0.73 aH-11→bL; 0.49 aH-10→bL; -0.21 bH-7→bL
620.6	2.00	0.02	0.49 bH-3→bL+1; -0.34 bH-5→bL+1; -0.32 bH-2→bL+2
618.5	2.01	0.03	-0.65 bH-3→bL; -0.28 bH-2→bL+3; 0.27 bH-4→bL+1
613.2	2.02	0.03	0.75 bH-2→bL+2; 0.21 aH-13→bL; 0.18 bH-5→bL
609.3	2.04	0.02	0.45 aH-12→bL; -0.44 bH-2→bL+3; 0.33 bH-5→bL
606.7	2.04	0.01	0.5 aH-12→bL; -0.48 aH-14→bL; -0.38 bH-3→bL
593.3	2.09	0.01	0.5 aH-13→bL; 0.3 bH-4→bL; 0.27 bH-2→bL+2
592.2	2.09	0.02	0.51 aH-14→bL; 0.39 bH-1→bL+5; 0.31 aH-12→bL
585.5	2.12	0.03	0.51 bH-1→bL+5; 0.46 bH-1→bL+4; 0.38 aH-13→bL
582.6	2.13	0.01	0.5 bH-2→bL+1; -0.33 bH-3→bL+2; -0.31 bH-1→bL+5
579.4	2.14	0.03	0.51 bH-1→bL+6; -0.47 bH-1→bL+5; -0.36 bH-2→bL+1
571.5	2.17	0.02	0.64 aH-15→bL; -0.34 bH-3→bL+1; -0.21 bH-6→bL
569.3	2.18	0.02	0.51 bH-3→bL+1; 0.46 aH-15→bL; 0.28 bH-5→bL+1
549.1	2.26	0.01	-0.46 bH-4→bL+1; 0.4 bH-6→bL+1; 0.38 bH-3→bL+1
531.3	2.33	0.04	0.47 bH-7→bL; 0.37 bH-2→bL+2; -0.27 bH-1→bL+5
530.6	2.34	0.14	-0.49 bH-7→bL; 0.41 bH-1→bL+6; 0.29 bH-1→bL+5
529.8	2.34	0.16	0.39 bH-1→bL+5; -0.32 bH-1→bL+4; 0.29 bH-1→bL+6
528.4	2.35	0.02	0.42 bH-7→bL+1; -0.37 bH-4→bL+2; -0.35 aH-8→bL

524.7	2.36	0.09	0.36 bH-7→bL; -0.31 bH-3→bL+2; -0.28 bH-2→bL+2
520.2	2.38	0.11	0.4 bH-5→bL+1; 0.4 aH-9→bL+1; -0.34 aH-8→bL
519.0	2.39	0.02	0.41 bH-4→bL+2; -0.4 aH-8→bL; -0.38 bH-5→bL+1
507.7	2.44	0.03	-0.47 aH-8→bL+1; -0.42 aH-9→bL; -0.31 aH-8→bL
504.7	2.46	0.02	-0.4 aH-10→bL; -0.39 aH-9→bL+1; 0.32 aH-8→bL+1
502.9	2.47	0.01	-0.62 bH-5→bL+2; 0.37 bH-3→bL+2; 0.24 aH-15→bL
501.4	2.47	0.02	0.4 aH-20→bL; -0.39 aH-15→bL; -0.39 bH-5→bL+2
498.4	2.49	0.02	-0.51 aH-10→bL; 0.35 aH-9→bL+1; -0.24 bH-6→bL
484.8	2.56	0.02	0.6 bH-6→bL+2; -0.39 bH-7→bL+1; 0.24 aH-9→bL+2
480.9	2.58	0.01	0.5 bH-6→bL+1; -0.35 bH-7→bL+2; -0.32 aH-10→bL+1
479.2	2.59	0.02	-0.52 aH-11→bL; -0.31 aH-22→bL; 0.28 aH-20→bL
477.4	2.60	0.06	-0.41 aH-19→bL; 0.36 aH-11→bL+1; -0.31 aH-17→bL
471.5	2.63	0.01	0.61 aH-9→bL+1; -0.36 bH-6→bL+1; 0.22 aH-16→bL
468.8	2.65	0.03	0.4 bH-3→bL+2; -0.29 aH-16→bL; -0.27 aH-11→bL
468.0	2.65	0.01	0.34 bH-3→bL+2; 0.28 aH-12→bL+1; -0.27 bH-4→bL+2
467.3	2.65	0.01	0.49 bH-3→bL+3; -0.32 aH-17→bL; 0.29 aH-21→bL
465.3	2.66	0.12	0.39 aH-10→bL+1; 0.28 aH-16→bL; 0.27 bH-3→bL+2
464.4	2.67	0.01	-0.45 bH-3→bL+3; 0.31 aH-10→bL+1; -0.24 aH-11→bL+1
463.2	2.68	0.01	0.36 aH-17→bL; -0.3 bH-3→bL+2; 0.29 aH-10→bL+1
461.3	2.69	0.02	-0.48 aH-18→bL; 0.32 aH-27→bL; -0.28 aH-23→bL
461.2	2.69	0.02	0.33 aH-18→bL; -0.28 aH-27→bL; -0.27 aH-16→bL
460.0	2.70	0.02	-0.34 bH-4→bL+3; -0.3 aH-12→bL; -0.26 bH-4→bL+2
459.2	2.70	0.06	0.39 bH-5→bL+2; 0.26 aH-16→bL; 0.26 aH-18→bL
458.6	2.70	0.06	0.31 aH-15→bL; -0.28 aH-26→bL; -0.28 aH-16→bL
456.4	2.72	0.07	-0.25 bH-7→bL+2; -0.25 aH-9→bL+1; 0.24 aH-12→bL
454.8	2.73	0.01	0.48 bH-4→bL+3; -0.36 bH-5→bL+2; -0.29 bH-4→bL+2
454.4	2.73	0.05	0.36 bH-6→bL+1; 0.31 aH-9→bL+1; 0.3 bH-7→bL+1
451.2	2.75	0.05	0.31 aH-12→bL+1; 0.3 bH-4→bL+2; -0.26 bH-5→bL+2
450.6	2.75	0.01	-0.41 bH→bL+7; -0.26 aH-10→bL+2; -0.24 bH→bL+8
448.5	2.76	0.09	-0.29 aH-15→bL+1; -0.28 aH-11→bL; -0.25 bH→bL+6
447.5	2.77	0.04	0.35 aH-13→bL; 0.27 aH-15→bL+2; -0.26 aH-15→bL
446.3	2.78	0.01	0.34 aH-13→bL; 0.27 aH-10→bL+2; -0.26 aH-15→bL+2
443.6	2.80	0.02	0.34 aH-10→bL+2; -0.34 aH-9→bL+2; -0.25 aH-15→bL+1
442.7	2.80	0.01	-0.47 aH-9→bL+2; -0.35 bH→bL+6; -0.26 aH-14→bL
442.3	2.80	0.01	0.41 bH-6→bL+2; 0.32 bH-6→bL+3; -0.28 aH-13→bL
437.3	2.84	0.02	0.39 aH-21→bL; 0.38 aH-26→bL; 0.32 aH-17→bL
437.2	2.84	0.01	-0.42 bH→bL+6; -0.3 bH→bL+7; 0.26 aH-14→bL

436.8	2.84	0.03	0.48 aH-25→bL; 0.25 aH-16→bL; 0.25 bH-6→bL+3
434.8	2.85	0.02	0.34 aH-14→bL+1; 0.29 aH-14→bL; -0.23 aH-18→bL
428.0	2.90	0.04	0.3 aH-15→bL+1; 0.24 aH-11→bL+1; 0.21 aH-15→bL
420.2	2.95	0.01	0.35 bH-2→bL+3; 0.32 bH-2→bL+5; 0.28 bH-3→bL+4
417.9	2.97	0.01	0.32 bH-4→bL+5; 0.27 bH-4→bL+3; 0.27 aH-9→bL+2
412.3	3.01	0.05	-0.5 aH-11→bL+1; 0.22 aH-19→bL; 0.21 bH-1→bL+6
411.1	3.02	0.01	-0.43 aH-11→bL+2; -0.24 bH-7→bL+2; 0.23 aH-19→bL+1
410.5	3.02	0.02	-0.45 aH-12→bL+1; 0.36 bH-7→bL+2; -0.33 bH-2→bL+4
408.4	3.04	0.03	0.33 bH-1→bL+6; -0.3 aH-12→bL+2; -0.23 aH-9→bL+3
407.6	3.04	0.02	-0.43 bH-7→bL+3; -0.29 bH-2→bL+3; -0.21 aH-13→bL+1
406.3	3.05	0.09	0.27 bH-3→bL+3; -0.25 bH-4→bL+4; 0.25 aH-8→bL+3
405.4	3.06	0.01	-0.31 aH-9→bL+2; -0.28 bH-5→bL+5; -0.26 bH-7→bL+2
404.3	3.07	0.04	0.27 bH-4→bL+5; 0.26 aH-8→bL+2; -0.26 bH-3→bL+3
403.8	3.07	0.05	-0.36 bH-3→bL+4; 0.32 bH-5→bL+4; -0.25 aH-9→bL+3
403.4	3.07	0.14	-0.43 bH-4→bL+4; 0.38 aH-14→bL+1; -0.24 bH-2→bL+4
401.6	3.09	0.07	-0.55 bH-2→bL+4; 0.25 bH-3→bL+3; -0.23 aH-19→bL+1
400.9	3.09	0.03	0.35 aH-14→bL+1; -0.29 bH-2→bL+5; 0.27 bH-2→bL+4

(1) Excitations with oscillator strength, $F_{osc.}$ above 0.01 are shown.

(2) H stands for HOMO, L stands for LUMO

Table S6. The calculated UV-vis-NIR absorption spectrum of ***g*-Y_2[(MeO)$_8$Pc]$_3$$^{2+}$** for the BP86/def2-SVP geometry calculated by the sTDA method at the CAM-B3LYP/6-31G(d) level of theory.

Wavelength, nm	Energy, eV	$F_{osc.}$ (1)	Wavefunction (2)
3137.3	0.40	0.51	0.97 H→L; 0.18 H-1→L; 0.04 H-26→L
1080.2	1.15	0.01	-0.97 H-5→L; -0.09 H-14→L; 0.08 H-4→L+6
1072.0	1.16	0.08	0.71 H-1→L+1; 0.58 H-3→L; -0.19 H→L+4
1071.9	1.16	0.08	-0.71 H-1→L+2; -0.58 H-4→L; -0.19 H→L+3
911.8	1.36	0.01	-0.93 H-8→L; 0.21 H-7→L; 0.16 H→L+4
911.8	1.36	0.01	-0.93 H-7→L; -0.21 H-8→L; 0.16 H→L+3
784.5	1.58	0.16	-0.54 H→L+4; -0.5 H→L+3; 0.42 H-1→L+3
784.5	1.58	0.16	0.54 H→L+3; -0.5 H→L+4; 0.42 H-1→L+4
759.4	1.63	0.02	-0.94 H-10→L; 0.25 H-14→L; -0.1 H-5→L
750.7	1.65	0.08	0.77 H-1→L+3; 0.42 H→L+3; -0.27 H-12→L
750.7	1.65	0.08	0.77 H-1→L+4; 0.42 H→L+4; 0.27 H-13→L
696.6	1.78	0.11	0.88 H-12→L; 0.22 H→L+3; 0.17 H-1→L+5
696.6	1.78	0.11	-0.88 H-13→L; 0.22 H→L+4; 0.17 H-1→L+6
631.8	1.96	0.01	-0.92 H→L+6; -0.18 H-1→L+4; -0.15 H→L+1
631.8	1.96	0.01	-0.92 H→L+5; -0.18 H-1→L+3; 0.15 H→L+2
595.2	2.08	0.03	0.83 H-2→L+1; 0.32 H-1→L+6; -0.28 H-5→L+2
595.2	2.08	0.03	-0.83 H-2→L+2; -0.32 H-1→L+5; 0.28 H-5→L+1
590.5	2.10	0.01	0.62 H-4→L+2; 0.61 H-3→L+1; 0.31 H-4→L+1
586.6	2.11	0.09	-0.75 H-1→L+5; 0.38 H-2→L+2; -0.3 H-15→L
586.6	2.11	0.09	-0.75 H-1→L+6; 0.38 H-2→L+1; 0.3 H-16→L
554.4	2.24	0.03	0.87 H-5→L+1; 0.27 H-2→L+2; 0.2 H-5→L+2
554.4	2.24	0.03	-0.87 H-5→L+2; -0.27 H-2→L+1; 0.2 H-5→L+1
525.5	2.36	0.11	-0.61 H-16→L; 0.38 H-6→L+1; -0.31 H-19→L
525.5	2.36	0.11	0.61 H-15→L; 0.37 H-6→L+2; 0.31 H-20→L
518.6	2.39	0.03	-0.83 H-6→L+2; 0.29 H-19→L; 0.21 H-23→L
518.6	2.39	0.03	0.83 H-6→L+1; -0.29 H-20→L; 0.21 H-16→L
508.6	2.44	0.41	-0.54 H-15→L; 0.54 H-20→L; 0.3 H-19→L
508.6	2.44	0.41	0.55 H-16→L; -0.54 H-19→L; 0.3 H-20→L
501.1	2.47	0.01	0.66 H-8→L+2; 0.66 H-7→L+1; -0.16 H-21→L
487.3	2.54	0.01	-0.72 H-24→L; -0.44 H-20→L; 0.34 H-23→L
487.3	2.54	0.01	0.72 H-23→L; -0.44 H-19→L; 0.34 H-24→L
478.2	2.59	0.23	0.78 H-9→L+2; -0.42 H-10→L+1; -0.15 H-9→L+1
478.2	2.59	0.23	-0.78 H-9→L+1; -0.42 H-10→L+2; -0.15 H-9→L+2
454.6	2.73	0.21	0.6 H-10→L+2; 0.5 H-11→L+1; -0.43 H-14→L+2

454.6	2.73	0.21	-0.6 H-10→L+1; -0.5 H-11→L+2; 0.43 H-14→L+1
444.4	2.79	0.01	-0.53 H-12→L+2; -0.53 H-13→L+1; -0.42 H-12→L+1
440.2	2.82	0.18	-0.63 H-11→L+2; -0.44 H-11→L+1; 0.39 H-10→L+2
440.2	2.82	0.18	0.62 H-11→L+1; -0.44 H-11→L+2; 0.39 H-10→L+1
432.0	2.87	0.05	0.78 H-2→L+4; 0.34 H-2→L+3; 0.24 H-5→L+4
432.0	2.87	0.05	-0.78 H-2→L+3; 0.34 H-2→L+4; 0.24 H-5→L+3
430.9	2.88	0.01	-0.39 H-13→L+2; 0.39 H-12→L+1; -0.36 H-4→L+3
430.4	2.88	0.04	0.55 H-4→L+3; -0.5 H-3→L+4; 0.31 H-12→L+1
412.9	3.00	0.23	0.61 H-5→L+3; 0.39 H-5→L+4; 0.29 H-2→L+6
412.9	3.00	0.23	-0.61 H-5→L+4; 0.39 H-5→L+3; -0.29 H-2→L+5
407.3	3.04	0.06	0.44 H-14→L+2; -0.36 H-5→L+4; -0.34 H-6→L+3
407.3	3.04	0.07	-0.44 H-14→L+1; 0.36 H-5→L+3; 0.34 H-6→L+4

(1) Excitations with oscillator strength, $F_{osc.}$ above 0.01 are shown.

(2) H stands for HOMO, L stands for LUMO

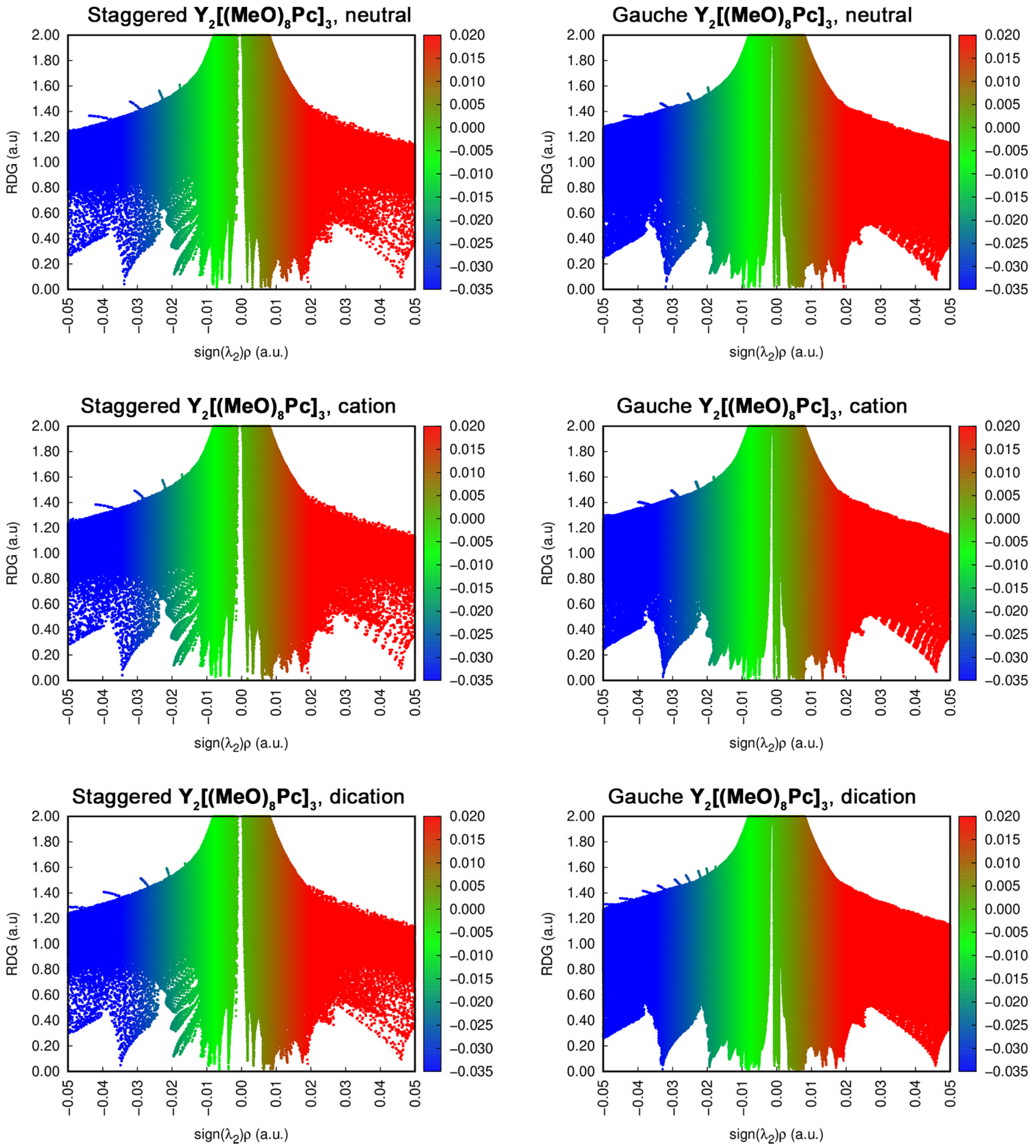

Figure S10. RDG vs. *sign*(λ_2)ρ plots for staggered and gauche conformations of redox-forms of **$Y_2[(MeO)_8Pc]_3$**.

Table S7. BP86/def2-SVP geometry of ***s*-Y₂[(MeO)₈Pc]₃**.

C	-4.19022374357489	-0.70694999595402	3.19212065920735
C	-4.19024149117518	0.70697895597704	3.19207007614298
C	-2.79408283831429	1.12459669558743	3.06073968948238
C	-2.79406429273401	-1.12455157324893	3.06080559154482
C	2.79385540487487	1.12458455790370	3.06089968289158
C	4.19001296826811	0.70697470962797	3.19222275058415
C	4.19003239823189	-0.70695381934481	3.19202373267347
C	2.79385889122695	-1.12456290963247	3.06085168257624
C	-1.12467475662178	-2.79393809761602	3.06093599652359
C	1.12447065079768	-2.79395094164203	3.06090008964808
C	0.70685603479697	-4.19010292548361	3.19229487849853
C	-0.70707279986007	-4.19008937370297	3.19235069894216
C	-0.70707946012578	4.19012764250965	3.19233308069000
C	0.70685148982098	4.19011085392174	3.19243292250279
C	1.12445821868947	2.79396535622395	3.06098058603429
C	-1.12469162590879	2.79398266565200	3.06084751960947
C	-1.43567052057089	-5.38571571855717	3.28998674034155
C	-0.72169125489876	-6.59124736989629	3.36330361777320
C	0.72143247575701	-6.59126304060962	3.36325721816585
C	1.43543436457268	-5.38574553917789	3.28989086416823
C	-5.38591454465207	1.43554398686599	3.28937819842589
C	-6.59144143720471	0.72153056200963	3.36249751152265
C	-6.59141549929537	-0.72158948184516	3.36256131944108
C	-5.38586237490538	-1.43555762235093	3.28949444532403
C	5.38568121691892	-1.43553783054905	3.28944099061871
C	6.59123941151716	-0.72154716220686	3.36225922315228
C	6.59125644632612	0.72156998220400	3.36196371608728
C	5.38569407055720	1.43556109793622	3.28922961824133
C	1.43545041208183	5.38574301934509	3.28996297372292
C	0.72147472408293	6.59128242985640	3.36318968795449
C	-0.72164225507247	6.59130536790187	3.36307550403911
C	-1.43565024479782	5.38578703772042	3.28976715121368
C	-2.68272018790727	-7.86733697687025	3.78032151215083
C	2.68248322190000	-7.86741131161268	3.77998578074335
C	7.86733544223236	-2.68247765766412	3.77967013751302
C	7.86724218895634	2.68248749920671	3.77978043671744
C	2.68243284968338	7.86726993209167	3.78082172862986
C	-2.68260695130446	7.86733093330858	3.78055833648869
C	-7.86753449774789	2.68248785408038	3.77980013793906
C	-7.86743783483532	-2.68257263626333	3.77995623654002
C	-2.44740831580725	-3.44546889438366	0.00013661227833
C	-3.44547182237745	-2.44739033929571	0.00005050159751
C	-2.75920122357768	-1.16870900955386	-0.00001629839522
C	-1.16871836309984	-2.75921091594537	0.00008441420822
C	1.16870651719267	2.75921041831966	0.00015433990460
C	2.44738911981005	3.44547680463482	0.00024501606866
C	3.44546281860786	2.44740962398157	0.00021881745551
C	2.75920022045778	1.16872183324357	0.00013410654258
C	1.16870713817114	-2.75920765538175	0.00012332044580
C	2.75919378718480	-1.16870751170482	0.00009487970522
C	3.44546267561807	-2.44739120704802	0.00005182999938
C	2.44739684729484	-3.44546749181229	0.00009448388143
C	-3.44547423288072	2.44740832489049	-0.00016167949861
C	-2.44740146762394	3.44547751296555	-0.00007975411167
C	-1.16871745958002	2.75921230991208	0.00002157479747
C	-2.75920956317861	1.16872017888421	-0.00006956310220
C	2.77491187074415	-4.80885628261553	0.00004660770821
C	4.13278617593572	-5.15635283142341	-0.00009257868229
C	5.15637268099394	-4.13275326211023	-0.00018454351573
C	4.80885535299095	-2.77488476146438	-0.00009373056032
C	-4.80886646549797	-2.77487497875765	0.00007544536842
C	-5.15639158517214	-4.13274103331875	0.00020499255553
C	-4.13280909106984	-5.15634438242100	0.00031173337994
C	-2.77493276866517	-4.80885561094612	0.00025565917317
C	4.80884962606789	2.77492435579172	0.00022632947514
C	5.15634816939311	4.13279849248891	0.00034590764502
C	4.13275007847437	5.15638589509075	0.00044018123580
C	2.77488160642704	4.80886886879203	0.00036806971571
C	-2.77490072957930	4.80886844495577	-0.00017071778103
C	-4.13277021712729	5.15638091400469	-0.00034812352625
C	-5.15636630383270	4.13279134397430	-0.00044953668364
C	-4.80886381507647	2.77491746560344	-0.00034318921984
C	3.61364480422054	-7.47218609852605	0.00000392240464
C	7.47219579611046	-3.61357023921068	-0.00056229788096
C	7.47218295846112	3.61365040996167	-0.00002542791320
C	3.61355413194797	7.47220535537777	0.00065807984116
C	-3.61358466932363	7.47220330278022	-0.00007530652023
C	-7.47219487859633	3.61363440472851	-0.00050185142467
C	-7.47221112974645	-3.61354489863591	0.00004440440013
C	-3.61367918692904	-7.47217897559722	0.00043347438944
C	-4.19001123915849	-0.70698018890046	-3.19230555822155
C	-4.18999333838446	0.70694853565772	-3.19237950456035
C	-2.79385039482957	1.12455100142733	-3.06090521111750
C	-2.79386894652962	-1.12459487868182	-3.06081530396011
C	2.79407720909807	1.12456480923909	-3.06066446700690
C	4.19021913863429	0.70695131977387	-3.19217590361525
C	4.19021843431091	-0.70697847469252	-3.19208722036053
C	2.79405987655828	-1.12457888319991	-3.06075575389312
C	-1.12447570444173	-2.79397721365621	-3.06076599932339
C	1.12466906440388	-2.79396339150196	-3.06077485758716
C	0.70706949152770	-4.19012073627325	-3.19210608065898
C	-0.70685814058162	-4.19013212480444	-3.19211047460528
C	-0.70685484510764	4.19009573789653	-3.19215635833179
C	0.70707473280094	4.19010787674617	-3.19205955888813
C	1.12468505882703	2.79395096954309	-3.06070866226266
C	-1.12446497153764	2.79394013251820	-3.06083216775544
C	-1.43543155310469	-5.38578334922837	-3.28962733334047
C	-0.72142501394060	-6.59130430782865	-3.36289569264487
C	0.72169948217247	-6.59128363177777	-3.36295637606161
C	1.43567355462731	-5.38574636497172	-3.28969338739512
C	-5.38562179916924	1.43555468989485	-3.28990610626618
C	-6.59116436236137	0.72158397172806	-3.36312240037793
C	-6.59118741180776	-0.72153733534139	-3.36305461505429
C	-5.38566996468101	-1.43554806688640	-3.28976691555751
C	5.38583490983947	-1.43557574684444	-3.28982786281645
C	6.59138697601230	-0.72159957745553	-3.36285372754210
C	6.59143313072595	0.72152014913086	-3.36238972159422
C	5.38589662417426	1.43552801765822	-3.28934825090651
C	1.43565198924145	5.38577213966196	-3.28933851469227
C	0.72165043228168	6.59130721553325	-3.36244510021211
C	-0.72146758893145	6.59129155942806	-3.36253877478574
C	-1.43544839081748	5.38574154516497	-3.28952256571425
C	-2.68246270231754	-7.86746134062009	-3.77965019245155
C	2.68274787688195	-7.86739470324756	-3.77980953138688
C	7.86740587279607	-2.68245802836558	-3.78088249790358
C	7.86738021258915	2.68241601072178	-3.78044276027642
C	2.68260827991202	7.86738339162683	-3.77979773846068
C	-2.68240539357181	7.86732812434242	-3.78010775720590
C	-7.86713415297747	2.68255880837317	-3.78072465491603
C	-7.86724574644756	-2.68250960071638	-3.78040638365585
H	-2.53269118474039	-5.35237484304037	3.29146401232844
H	2.53245577310072	-5.35242448183239	3.29135719782338
H	-5.35262257468922	2.53256673721153	3.29069170048068
H	-5.35251471877712	-2.53257819671285	3.29089566351564
H	5.35236972390317	-2.53255854873171	3.29065111168045
H	5.35245042167497	2.53258343166259	3.28955098749523
H	2.53247144344972	5.35240352310184	3.29140805309614
H	-2.53267322733145	5.35249655415195	3.29100916145679
H	-2.89399247410781	-7.32628342693694	4.72978802370921
H	-3.32097250034066	-7.42996052976739	2.98027535887973
H	-2.94157204119735	-8.93596438417835	3.89882269437169
H	2.89393061203417	-7.32636162761288	4.72941576937380
H	3.32061911199387	-7.43005722373177	2.97983360733953
H	2.94131994365920	-8.93604651268947	3.89844913017622
H	8.93597385193375	-2.94134438881117	3.89803833765440
H	7.42979220070178	-3.32094910619293	2.97988680419657
H	7.32642430772352	-2.89343925079641	4.72928549830307
H	8.93585243966248	2.94135606402120	3.89840036982069
H	7.42986657678109	3.32102327739038	2.97995535222890
H	7.32613310204550	2.89336337609768	4.72930051809471
H	2.94128435271184	8.93587465641161	3.89953248216369
H	3.32089624941988	7.43000918702825	2.98087899292607
H	2.89342768185375	7.32605265294531	4.73025434876824
H	-2.94143050443825	8.93593424468873	3.89934383851016
H	-3.32097232761937	7.43018266889237	2.98047420054836
H	-2.89376714193132	7.32602342833604	4.72990297838520

H	-7.32642586431528	2.89364196525952	4.72926002796487
H	-7.43021627686590	3.32085005664575	2.97980682315721
H	-8.93615829540700	2.94131340133421	3.89839440617339
H	-7.32639923892487	-2.89360651668986	4.72948294635884
H	-7.43001209982648	-3.32098081351212	2.98005995353700
H	-8.93605715290914	-2.94144576173221	3.89848437897732
H	1.97345520700759	-5.55713188042821	0.00009182954319
H	5.55711939298735	-1.97341788716889	-0.00017319273340
H	-5.55712358946331	-1.97340122596146	-0.00000590150245
H	-1.97348382390645	-5.55713940028751	0.00034889398732
H	5.55712778706858	1.97347134130863	0.00016847378910
H	1.97341508673404	5.55713342443232	0.00043009094747
H	-1.97343970733933	5.55713878232026	-0.00009799062786
H	-5.55713695332231	1.97345859818063	-0.00043294472943
H	2.96015647732355	-7.42679486896936	0.89850157102598
H	2.95983284026148	-7.42686079822711	-0.89826090316202
H	4.17026711904783	-8.42725415659883	-0.00006451309033
H	7.42701782954673	-2.95993299916918	0.89783880478418
H	7.42663109571705	-2.95991294444854	-0.89892861715344
H	8.42727331738054	-4.17017647913040	-0.00077313396742
H	8.42725126693396	4.17027279604544	-0.00006411598077
H	7.42666102414297	2.96021050599271	-0.89855294771949
H	7.42698813765921	2.95979528559235	0.89821458985037
H	4.17015296035358	8.42728668790829	0.00081946240048
H	2.95994641570740	7.42696430160013	-0.89776100558128
H	2.95986895638864	7.42669516934738	0.89900450348949
H	-4.17018832744338	8.42728182526883	-0.00006672731726
H	-2.95969257304051	7.42699518467780	-0.89828895197690
H	-2.96018315169000	7.42666873342778	0.89847684788637
H	-8.42726777125566	4.17024776173086	-0.00055771186890
H	-7.42683847596573	2.95988920819311	-0.89881501166982
H	-7.42681862316864	2.96008317520623	0.89795080863275
H	-7.42689548403141	-2.95972643130997	0.89830543832997
H	-7.42677610726820	-2.96006799020729	-0.89845933600832
H	-8.42729216367637	-4.17014515227557	0.00009175437636
H	-2.95999174844802	-7.42676134899262	0.89878444039941
H	-2.96006503193460	-7.42688522469880	-0.89797825371021
H	-4.17030486723317	-8.42724528980974	0.00052075229761
H	-2.53245284591626	-5.35247131401752	-3.29101682325131
H	2.53269354160010	-5.35239287418661	-3.29124232045389
H	-5.35227363868376	2.53257542877215	-3.29132514171891
H	-5.35237501583826	-2.53257047899970	-3.29106618920655
H	5.35250833687167	-2.53259675838654	-3.29119626249355
H	5.35267970143621	2.53255224262006	-3.28954094591550
H	2.53267439831955	5.35247385522680	-3.29062569680837
H	-2.53246951946895	5.35241150441315	-3.29096948823345
H	-3.32062711639201	-7.43006629187854	-2.97954330313890
H	-2.89386668455257	-7.32645223791066	-4.72911321579180
H	-2.94130213367816	-8.93610013322170	-3.89807588298531
H	3.32097843651535	-7.42993991655675	-2.97978945563616
H	2.89403229398561	-7.32642387637353	-4.72931997818024
H	2.94161172809143	-8.93603028297535	-3.89820917744361
H	8.93602079455883	-2.94127791824601	-3.89956336215726
H	7.32629473701162	-2.89319331336210	-4.73043461494519
H	7.43007932809199	-3.32113776907354	-2.98114772550208
H	8.93596489453362	2.94123109220970	-3.89940524795615
H	7.32597777931353	2.89338652602945	-4.72977601702448
H	7.43032014041129	3.32092738582186	-2.98042403466382
H	2.94144102811312	8.93600624117716	-3.89837757425214
H	2.89364676458287	7.32629135592689	-4.72929338268616
H	3.32104696444947	7.43005551191534	-2.97987139379279
H	-2.94125429885205	8.93594865087077	-3.89867315217305
H	-2.89328574028136	7.32627611232104	-4.72966114584536
H	-3.32094788391107	7.42994066927782	-2.98029772752687
H	-7.42984604078828	3.32097568874461	-2.98075990106262
H	-7.32594092408700	2.89359156908669	-4.73016377063191
H	-8.93573641891488	2.94141934345571	-3.89943274844017
H	-7.43001073125902	-3.32083989820431	-2.98034252493637
H	-7.32604276370747	-2.89370524957389	-4.72980360466320
H	-8.93585884206818	-2.94133295330961	-3.89910006290335
N	-1.99887283639732	0.00002298023294	2.92340525324838
N	1.99865526086796	0.00001212319291	2.92352870878338
N	-0.00010064005497	-1.99876098794972	2.92346093819525
N	-0.00011427117778	1.99878764806896	2.92347782376924
N	-2.39946109200766	-2.39933434031848	3.09391507782121
N	-2.39948189432390	2.39938270802657	3.09379486373004
N	2.39924542680924	2.39936449908418	3.09401224299909
N	2.39926128078677	-2.39935250301797	3.09386381690804
N	-1.37631664591368	-1.37632250115782	0.00002025369096
N	1.37631228915794	1.37632495666648	0.00011604148006
N	1.37630784603961	-1.37631908029583	0.00014140630020
N	-1.37632214539881	1.37632614225406	0.00004312227833
N	-0.00000484810756	-3.39420924220700	0.00012669448909
N	-3.39420306795482	0.00000450485021	-0.00007398863403
N	-0.00000509330345	3.39421101457975	0.00010301853862
N	3.39419376873714	0.00000655666499	0.00011429666039
N	-1.99867282310244	-0.00002165413451	-2.92341523047307
N	1.99887954157105	-0.00000785678335	-2.92327399241041
N	0.00009403263825	-1.99878145289234	-2.92333326377604
N	0.00010480897905	1.99874957141020	-2.92338090668343
N	-2.39926362723267	-2.39938053961434	-3.09380801520261
N	-2.39924637370050	2.39933515176380	-3.09394720113042
N	2.39947386671153	2.39935123751741	-3.09370014540422
N	2.39945487900866	-2.39936285395399	-3.09379925875681
O	-1.30321837759655	-7.82242959443663	3.45490375632523
O	1.30293206701697	-7.82246051593364	3.45479358057136
O	7.82242543984710	-1.30307150138222	3.45384432260011
O	7.82241940097981	1.30310526133406	3.45382571256040
O	1.30301829024045	7.82244330144223	3.45501527518247
O	-1.30315088316225	7.82247754855157	3.45494494842790
O	-7.82262813262682	1.30303299763061	3.45418635069838
O	-7.82258315541622	-1.30313914968393	3.45422912918621
O	4.58906851013346	-6.43979119907156	-0.00019881349466
O	6.43981884271223	-4.58901487175321	-0.00036501309554
O	6.43978771570887	4.58907741848892	0.00036361326565
O	4.58900699386291	6.43983320364798	0.00054488130534
O	-4.58903160855171	6.43982634879786	-0.00050471305927
O	-6.43980692019599	4.58906694896179	-0.00060452113346
O	-6.43983959651833	-4.58899589648822	0.00028638863990
O	-4.58909772470841	-6.43978073687634	0.00039905989593
O	-1.30292392798141	-7.82250469941755	-3.45440127894931
O	1.30323670751726	-7.82247153145581	-3.45442654064810
O	7.82255043230001	-1.30313275353992	-3.45469755875127
O	7.82258538080924	1.30302059907296	-3.45455129430854
O	1.30317914518451	7.82249205304819	-3.45404794421643
O	-1.30301949655839	7.82246551009208	-3.45416045345591
O	-7.82232033748144	1.30312685266012	-3.45498406104800
O	-7.82236641287235	-1.30303926771526	-3.45485041033560
Y	-0.00006788028021	0.00001376858297	1.70131735226762
Y	0.00005688735274	-0.00000350089957	-1.70121407132855

Table S8. BP86/def2-SVP geometry of ***s*-Y$_2$[(MeO)$_8$Pc]$_3^+$**.

C	-4.19205689394472	-0.70670659425200	3.16067110350712
C	-4.19205776308625	0.70664883869617	3.16066286307295
C	-2.79478069757771	1.12433664975361	3.02842688030112
C	-2.79478441295441	-1.12439699407973	3.02840721332501
C	2.79428139908240	1.12434276429500	3.02877919550720
C	4.19157451163487	0.70665698351394	3.16083766626771
C	4.19155348923577	-0.70669842549311	3.16108256970939
C	2.79429632724664	-1.12439602162629	3.02870100093809
C	-1.12461540673505	-2.79456705381915	3.02853232484613
C	1.12412203029568	-2.79456227398328	3.02869274430266
C	0.70642299821579	-4.19183385013702	3.16093589114300
C	-0.70693144547444	-4.19183748845170	3.16082244732386
C	-0.70693381015136	4.19180084860298	3.16056370381138
C	0.70642174110255	4.19181218044288	3.16060270487347
C	1.12412400413007	2.79452302531970	3.02853856780600
C	-1.12461047353348	2.79451153118875	3.02844831609002
C	-1.43684293241613	-5.38480998332676	3.26723113873617
C	-0.72243946558510	-6.59144276158580	3.34977081641255
C	0.72190934590599	-6.59144202076916	3.34986610178912
C	1.43632214626973	-5.38480659007227	3.26743228107741
C	-5.38501586403484	1.43655018349085	3.26733089650809
C	-6.59163133264028	0.72213658120102	3.35004941648650
C	-6.59163012762011	-0.72221030774879	3.35003816005066
C	-5.38501039156106	-1.43661643683914	3.26731902963194
C	5.38454406502253	-1.43660900628240	3.26724624164614
C	6.59117227606209	-0.72220391964596	3.34983146841430
C	6.59114976492630	0.72213962961160	3.35015599097771
C	5.38452606471970	1.43655210277267	3.26755836016675
C	1.43630888285853	5.38479577774809	3.26703055322973
C	0.72188444722692	6.59142840799474	3.34942689581010
C	-0.72245707329416	6.59141290823237	3.34939522363968
C	-1.43685124733203	5.38476816392610	3.26695183678802
C	-2.67934047279034	-7.87003425106674	3.79180883026807
C	2.67876143915674	-7.87004153400318	3.79209905461755
C	7.86978367907088	-2.67917878624456	3.79147392093263
C	7.86983794555651	2.67902374885556	3.79201014055523
C	2.67873554874773	7.87011579030206	3.79143606493939
C	-2.67933817595463	7.87004904762848	3.79141827871279
C	-7.87023408527060	2.67899233282953	3.79228580463136
C	-7.87022518942614	-2.67909511439519	3.79216616463242
C	-2.44912710170178	-3.44688415082319	0.00007120581105
C	-3.44686528999188	-2.44912781889474	0.00006973367518
C	-2.75887473678312	-1.16912440532254	-0.00003304604642
C	-1.16911134084711	-2.75890604156799	0.00001901938986
C	1.16911202142189	2.75885119540086	0.00025891019462
C	2.44911536339375	3.44684243437056	0.00041087366864
C	3.44687132987714	2.44910426908145	0.00046167435193
C	2.75889271740752	1.16908992835571	0.00036021058273
C	1.16910889841264	-2.75890024014460	0.00021865614461
C	2.75887947900461	-1.16912659115549	0.00034764877414
C	3.44686551523679	-2.44913118151840	0.00045852106034
C	2.44912232351495	-3.44688145324653	0.00039726049910
C	-3.44685926102884	2.44910291180405	0.00015868222530
C	-2.44910806814046	3.44684580066682	0.00017389288385
C	-1.16910347349523	2.75885619276088	0.00007903386208
C	-2.75887941705874	1.16909039451772	0.00000207721708
C	2.77379000069385	-4.80959827465704	0.00042544210028
C	4.13359838258808	-5.15778092675552	0.00050359982531
C	5.15778936039212	-4.13357827951730	0.00055476958481
C	4.80958638471203	-2.77377743959107	0.00053857406291
C	-4.80958944456853	-2.77375798226581	0.00015655692705
C	-5.15780660354506	-4.13355525889623	0.00022967956765
C	-4.13362458349365	-5.15776520762661	0.00018702184793
C	-2.77381215760377	-4.80959728820999	0.00011738794989
C	4.80958612208801	2.77378572346946	0.00047055992600
C	5.15775703240732	4.13359813142690	0.00040974605342
C	4.13354691478546	5.15778180244056	0.00038444003903
C	2.77374835732473	4.80956600517951	0.00039198293943
C	-2.77375945163569	4.80956534665837	0.00027008413339
C	-4.13356252318045	5.15776477451122	0.00038204839436
C	-5.15776384699187	4.13357339854498	0.00041689114788
C	-4.80957688020236	2.77376592159831	0.00029317188576
C	3.62172187639943	-7.47752028133798	0.00034797607609
C	7.47752026890604	-3.62165283952837	0.00031901852086
C	7.47749536128936	3.62172142686373	0.00025770681181
C	3.62159344772330	7.47750664227047	0.00027408597478
C	-3.62163748915894	7.47749549790436	0.00036999133880
C	-7.47750131168106	3.62167971396240	0.00086195250040
C	-7.47753244029660	-3.62161259037432	0.00062121466438
C	-3.62177760098287	-7.47751269763319	0.00008773038355
C	-4.19155174002897	-0.70667821954266	-3.16099617350716
C	-4.19154630926482	0.70667416755825	-3.16097723772591
C	-2.79427488629598	1.12436035650450	-3.02873177798131
C	-2.79428375419281	-1.12437537698465	-3.02874473460126
C	2.79476640475784	1.12435296774705	-3.02853040366903
C	4.19204657916580	0.70668214056986	-3.16071574041453
C	4.19204470619786	-0.70667150734167	-3.16094994010781
C	2.79480118785789	-1.12437720262442	-3.02843138874519
C	-1.12411197130237	-2.79453648826463	-3.02876378184241
C	1.12462286357524	-2.79453176134185	-3.02864491345352
C	0.70694135440593	-4.19178061306423	-3.16114997230838
C	-0.70641145337421	-4.19179095766061	-3.16116710595770
C	-0.70641361657398	4.19180364573951	-3.16087715471414
C	0.70693947537572	4.19179593008972	-3.16079320096641
C	1.12461531149226	2.79452765373705	-3.02850491180231
C	-1.12411331551536	2.79453271188175	-3.02863307293882
C	-1.43630483658999	-5.38474579037684	-3.26791000299585
C	-0.72188544466026	-6.59136152646239	-3.35057830357635
C	0.72246351428615	-6.59135985438183	-3.35041761913081
C	1.43686033350738	-5.38473567088771	-3.26770329582314
C	-5.38450515747754	1.43658782739251	-3.26747682947738
C	-6.59114285190390	0.72218814871695	-3.34999379342175
C	-6.59115074367850	-0.72215703255514	-3.34999377470450
C	-5.38452232286409	-1.43657347395504	-3.26749525380961
C	5.38503624516721	-1.43655655389710	-3.26733525137188
C	6.59162824551161	-0.72212085296187	-3.35021852413201
C	6.59157614432831	0.72222491075578	-3.35057741761229
C	5.38495641582575	1.43661015710620	-3.26769884718245
C	1.43686489852659	5.38474554855647	-3.26732586001494
C	0.72247363800699	6.59137879952345	-3.34996382063306
C	-0.72186971901005	6.59139300943334	-3.35004726505162
C	-1.43629908509797	5.38477359054749	-3.26748014323208
C	-2.67874883855102	-7.86993549965967	-3.79284591690493
C	2.67937738673064	-7.86986623538541	-3.79265204850293
C	7.87021863270048	-2.67903203985653	-3.79219720902331
C	7.87015117859681	2.67910314716609	-3.79278197449010
C	2.67935685557484	7.86994876601364	-3.79214642343032
C	-2.67869620954630	7.87003291882458	-3.79227670364546
C	-7.86977493955115	2.67912872880550	-3.79173702589200
C	-7.86979647825373	-2.67906043212037	-3.79186364665523
H	-2.53374714459742	-5.35236902890040	3.27637682067691
H	2.53322529748986	-5.35236547401975	3.27669323792797
H	-5.35256188380743	2.53345302410035	3.27680472710252
H	-5.35254482550711	-2.53351851281840	3.27678873874564
H	5.35205771560780	-2.53350896260915	3.27691681069007
H	5.35201119017294	2.53344561411766	3.27785002088038
H	2.53321158887233	5.35235133465370	3.27650885522843
H	-2.53375352220942	5.35229906911775	3.27639226183599
H	-2.88504125025502	-7.32309119799700	4.73815381698853
H	-3.32428560288731	-7.44491269345564	2.99108884333942
H	-2.92722587639104	-8.93927874262912	3.92148604664778
H	2.88437346849697	-7.32311169306104	4.73847110844130
H	3.32378447060636	-7.44491084929357	2.99144648052648
H	2.92663220007651	-8.93928811812289	3.92178660858546
H	8.93904845756659	-2.92711161237408	3.92089676248977
H	7.44452488394150	-3.32405243867717	2.99076907560737
H	7.32296480797127	-2.88494844358499	4.73787393172857
H	8.93911682234942	2.92689050195697	3.92145014379584
H	7.44458293803641	3.32410181817709	2.99146580674906
H	7.32305457247426	2.88459162163338	4.73847434022168
H	2.92659182418159	8.93939499786988	3.92089004794164
H	3.32385362790565	7.44486371809102	2.99092176784288
H	2.88426213879043	7.32333902421500	4.73791243504640
H	-2.92722066047035	8.93932196723005	3.92087514961909

H	-3.32447352931555	7.44477465924722	2.99093072182807
H	-2.88480916200211	7.32327134506969	4.73790578867296
H	-7.32313008335356	2.88461152889060	4.73855467874424
H	-7.44530220238316	3.32405806799277	2.99156206954510
H	-8.93946912207964	2.92680803892052	3.92218124657739
H	-7.32316678889810	-2.88477099791697	4.73844951701833
H	-7.44523855806185	-3.32409554651564	2.99141999713654
H	-8.93946253713441	-2.92693619031217	3.92199119156423
H	1.97381381387713	-5.55929360536352	0.00034360855289
H	5.55927195034847	-1.97379175854671	0.00055957534802
H	-5.55926252802738	-1.97376092799492	0.00018814920253
H	-1.97384822547800	-5.55930615594986	0.00012124007547
H	5.55929406933161	1.97382075651837	0.00050155762717
H	1.97375435400337	5.55924193763497	0.00034530935018
H	-1.97377864771074	5.55925563767205	0.00028563679996
H	-5.55927142760603	1.97378856620750	0.00033410321073
H	2.96996858952487	-7.43582811757083	0.89966741083345
H	2.97017262028806	-7.43576281058818	-0.89911739044228
H	4.18739176551577	-8.42651639353366	0.00037819693109
H	7.43585871079971	-2.96986069137322	0.89960716975115
H	7.43571140177085	-2.97014108429824	-0.89916967372472
H	8.42652687180913	-4.18730462025553	0.00033308168020
H	8.42649252526793	4.18739039121126	0.00023582011291
H	7.43574166439821	2.97010135251326	-0.89915520091559
H	7.43580845271713	2.97005115288059	0.89963517864585
H	4.18723515841757	8.42651997129355	0.00030677303508
H	2.96996052260959	7.43579865637304	-0.89912924682787
H	2.96993700037168	7.43573502158886	0.89965595783531
H	-4.18729019237046	8.42650237343924	0.00053305263799
H	-2.97014586321306	7.43583297962234	-0.89913894471001
H	-2.96983722209753	7.43569433242301	0.89964645789747
H	-8.42650089687844	4.18734478766274	0.00115809253657
H	-7.43607510276924	2.97003099445381	-0.89854345646554
H	-7.43547943699519	2.97003494686151	0.90024300386850
H	-7.43552637659813	-2.96997947117439	0.90001137787489
H	-7.43605583976992	-2.96994375920867	-0.89876853258135
H	-8.42654250575079	-4.18725846608696	0.00087859289560
H	-2.97002044289988	-7.43581374861323	0.89940427479201
H	-2.97022939316486	-7.43578179269658	-0.89937986359347
H	-4.18746027259762	-8.42650120821650	0.00013688424820
H	-2.53320658689941	-5.35229552194296	-3.27732747893379
H	2.53376572520462	-5.35229641753308	-3.27670546599958
H	-5.35203079656983	2.53348845348443	-3.27695332118494
H	-5.35206952244511	-2.53347488714927	-3.27695850397019
H	5.35259381939232	-2.53345823235643	-3.27700293577101
H	5.35241527435179	2.53350232406422	-3.27803405658022
H	2.53376724231733	5.35228398700398	-3.27669716205123
H	-2.53320092739571	5.35233882461366	-3.27701129610642
H	-3.32372539798642	-7.44497410415879	-2.99206502084794
H	-2.88444703977400	-7.32283752759749	-4.73910224966750
H	-2.92659590332138	-8.93916593992419	-3.92271263903828
H	3.32430072627977	-7.44493617631683	-2.99181194119647
H	2.88510935253781	-7.32270842902631	-4.73886589912946
H	2.92725595129928	-8.93908343579156	-3.92256706325970
H	8.93943856102987	-2.92687347582064	-3.92217919670166
H	7.32296051313707	-2.88481825433211	-4.73833582921718
H	7.44547814528809	-3.32396170213639	-2.99125960824921
H	8.93938135714016	2.92695455289517	-3.92266143830154
H	7.32302588875808	2.88461879119055	-4.73905661873354
H	7.44526337565678	3.32421985450028	-2.99207474044696
H	2.92723837981268	8.93918396507128	-3.92192501862389
H	2.88483320022819	7.32291538596979	-4.73848171136758
H	3.32447533601643	7.44495506998092	-2.99149655227948
H	-2.92653605239435	8.93927730018014	-3.92205931157196
H	-2.88419481702359	7.32300561636938	-4.73861132392727
H	-3.32383578225980	7.44506693750004	-2.99162781768688
H	-7.44452304658666	3.32405067114460	-2.99106895795159
H	-7.32296642242266	2.88482670815016	-4.73816025720763
H	-8.93903491525220	2.92703276759721	-3.92125151260132
H	-7.44460518720022	-3.32404421596329	-2.99121227856928
H	-7.32293889299274	-2.88470784894362	-4.73826917261347
H	-8.93905432630083	-2.92693573187329	-3.92145295993802
N	-1.99786355791077	-0.00003223271356	2.89652521564399
N	1.99736874981121	-0.00003072591158	2.89687938224072
N	-0.00024123855213	-1.99765029626928	2.89669912580486
N	-0.00023344954621	1.99758634106494	2.89669487463807
N	-2.39957629277539	-2.39935540584380	3.06032754971512
N	-2.39957033196710	2.39929659791934	3.06028249049956
N	2.39907899354260	2.39930971098895	3.06051152850960
N	2.39908017716323	-2.39935177756038	3.06062156076548
N	-1.37538010221394	-1.37540522023918	-0.00002743906818
N	1.37539252347225	1.37535667719410	0.00027818556161
N	1.37538154309122	-1.37540165016695	0.00023865783604
N	-1.37538088377401	1.37535848832550	0.00000702967441
N	-0.00000001929311	-3.39431648780267	0.00015127311166
N	-3.39429346246167	-0.00001704520442	0.00000661792528
N	0.00000499759025	3.39426720746640	0.00021386605969
N	3.39429969238028	-0.00002084131037	0.00043917775430
N	-1.99736734251196	-0.00001206900313	-2.89679880262366
N	1.99787820989847	-0.00001989495771	-2.89647724676471
N	0.00024671903999	-1.99764014786403	-2.89664744356791
N	0.00024508490629	1.99761748065279	-2.89662196469494
N	-2.39906680773476	-2.39932633699913	-3.06070398508584
N	-2.39906512491430	2.39931720751861	-3.06060583229887
N	2.39956382679112	2.39930949767597	-3.06040636462155
N	2.39958063507392	-2.39932276144038	-3.06047950325421
O	-1.29978547153783	-7.81730795009906	3.45526470131828
O	1.29924005368749	-7.81730835765881	3.45542198701606
O	7.81705022197258	-1.29957791930173	3.45510300894290
O	7.81704389932696	1.29950620150282	3.45531522060182
O	1.29923387650658	7.81732120545884	3.45467284502993
O	-1.29984466242729	7.81729344535377	3.45460494417087
O	-7.81749870774464	1.29947727763883	3.45557407339726
O	-7.81749504192223	-1.29955534464163	3.45555430923784
O	4.58998466282074	-6.43502318105166	0.00049004645210
O	6.43503937937859	-4.58993958020825	0.00053249305324
O	6.43499811819400	4.58998736049351	0.00032570990762
O	4.58989471695403	6.43503636319648	0.00026462387304
O	-4.58992442172908	6.43501454814544	0.00045748360314
O	-6.43500836271383	4.58995156314070	0.00054813907435
O	-6.43505993536845	-4.58990714857779	0.00031607540593
O	-4.59002465127022	-6.43500265437915	0.00021221295961
O	-1.29920616529263	-7.81722147189126	-3.45625700156410
O	1.29981388824419	-7.81720419799633	-3.45613105212823
O	7.81750153377469	-1.29945491477583	-3.45572495469494
O	7.81744036344681	1.29960786843178	-3.45596369437690
O	1.29986308854306	7.81724620424095	-3.45530446097450
O	-1.29921148844757	7.81727676745883	-3.45542499816445
O	-7.81702364196722	1.29956051133853	-3.45522705250164
O	-7.81703669375979	-1.29951537064978	-3.45525987077301
Y	-0.00014144262179	-0.00002242059127	1.69268191418957
Y	0.00014808277808	-0.00001218747869	-1.69258795229889

Table S9. BP86/def2-SVP geometry of ***s*-Y$_2$[(MeO)$_8$Pc]$_3$$^{2+}$**.

C	-4.19274024229602	-0.70657411062013	3.13806419680171
C	-4.19274287070403	0.70649473709438	3.13805139918995
C	-2.79507408698898	1.12406804478132	3.00123418444191
C	-2.79507573635538	-1.12414902607098	3.00121835740026
C	2.79498077092632	1.12406988122476	3.00138604433403
C	4.19265631438316	0.70650243598528	3.13814840672725
C	4.19263694725876	-0.70656675947102	3.13836031904524
C	2.79499733584489	-1.12414846499743	3.00128865999684
C	-1.12415197478304	-2.79507575963017	3.00127837392850
C	1.12406659001916	-2.79506893607814	3.00133802063215
C	0.70649249600011	-4.19272679415250	3.13823615122337
C	-0.70657655239672	-4.19273314154061	3.13819542336791
C	-0.70657885072998	4.19267299411440	3.13799116878182
C	0.70648997516678	4.19267480683669	3.13799542842343
C	1.12406616961122	2.79500250984681	3.00123321248007
C	-1.12414909866374	2.79499861799052	3.00121762151040
C	-1.43770247845786	-5.38270142876205	3.25832813988052
C	-0.72292966004488	-6.59028586362596	3.35560543927989
C	0.72286214731150	-6.59027527777935	3.35567411501530
C	1.43762552677207	-5.38268378555932	3.25843249032319
C	-5.38270454291978	1.43761905915076	3.25825024810406
C	-6.59029145854066	0.72284623845179	3.35549713532291
C	-6.59028900486251	-0.72294238849470	3.35549061948610
C	-5.38269706965219	-1.43770720053772	3.25824790911168
C	5.38261122306073	-1.43770141007225	3.25834661525978
C	6.59018408522946	-0.72293387234681	3.35578408924918
C	6.59016169656199	0.72285333690236	3.35603645457980
C	5.38258981793095	1.43762475979313	3.25861760190276
C	1.43761622806056	5.38264125734890	3.25810412390303
C	0.72284693718776	6.59023934564991	3.35523163958246
C	-0.72293792545687	6.59023841902746	3.35522937307370
C	-1.43770646434545	5.38263975714689	3.25809403858497
C	-2.67773845431128	-7.87101550309571	3.81622018506410
C	2.67765646315123	-7.87100323956005	3.81635861433314
C	7.87097145515375	-2.67782443036581	3.81590964566894
C	7.87089459460610	2.67762497184631	3.81682615223095
C	2.67762842316803	7.87104449980026	3.81578465593672
C	-2.67771774670178	7.87103792603682	3.81580360755464
C	-7.87107771656692	2.67763675302121	3.81604743723278
C	-7.87106713465648	-2.67775729583006	3.81595784446096
C	-2.45060726531514	-3.44849021362741	0.00007303573221
C	-3.44847470768534	-2.45060527147652	0.00008543339295
C	-2.75938873946371	-1.16976741185679	0.00003833608278
C	-1.16975742793775	-2.75941654359696	0.00003661488258
C	1.16975737057183	2.75936967421480	0.00011937581897
C	2.45059547891929	3.44845554819731	0.00019603374368
C	3.44847917340064	2.45058706397228	0.00021794999053
C	2.75940357017674	1.16973904990739	0.00014470992663
C	1.16975577375535	-2.75941334030678	0.00009443055260
C	2.75939477291391	-1.16977063457454	0.00013825520059
C	3.44847702458261	-2.45061057796561	0.00027014217457
C	2.45060451482687	-3.44849014418717	0.00018212269465
C	-3.44846726203004	2.45058359793629	0.00012340822913
C	-2.45058840601216	3.44845703067053	0.00011562464490
C	-1.16974863930705	2.75937340509114	0.00007974522265
C	-2.75939001990152	1.16973871597708	0.00005979517984
C	2.77316695581811	-4.81084916789918	0.00033770185878
C	4.13477183779588	-5.15978409882735	0.00044293658920
C	5.15979510938544	-4.13474853440634	0.00032386620580
C	4.81083967506893	-2.77315116809677	0.00026935025138
C	-4.81084062290727	-2.77313167323954	0.00009466231265
C	-5.15980838118974	-4.13472627268160	0.00008937183436
C	-4.13479235334584	-5.15976838083886	0.00006763252381
C	-2.77318428234009	-4.81084561305488	0.00007308420315
C	4.81083652132408	2.77315970903324	0.00025777307351
C	5.15976215512392	4.13476749271856	0.00025285763609
C	4.13472142382990	5.15978515389354	0.00022581763678
C	2.77312612221450	4.81082016617808	0.00020493262061
C	-2.77313691305956	4.81081838720708	0.00012016090743
C	-4.13473732171347	5.15976739823375	0.00011214308932
C	-5.15976954140033	4.13474112286103	0.00013609216726
C	-4.81082740932718	2.77313820237918	0.00014296453088
C	3.63240923672143	-7.48437921043074	0.00022444778242
C	7.48438122189407	-3.63233620986199	0.00049538874316
C	7.48435117199174	3.63239608321150	0.00038722697516
C	3.63228501979353	7.48436479138904	0.00014967362021
C	-3.63232816209876	7.48435304338599	0.00012829619324
C	-7.48435958316075	3.63235211277451	0.00014828481188
C	-7.48438981668860	-3.63229708549058	0.00005369938181
C	-3.63245273868230	-7.48437007914549	0.00008004335528
C	-4.19263562726188	-0.70652114283682	-3.13828397389862
C	-4.19263115200758	0.70654575720126	-3.13825116689370
C	-2.79497367823312	1.12411242151133	-3.00133229442663
C	-2.79498117613352	-1.12410125814329	-3.00136657132457
C	2.79506526487836	1.12411193533455	-3.00132162743005
C	4.19274158091515	0.70655586834306	-3.13807803704071
C	4.19273462061178	-0.70651285182520	-3.13831678296514
C	2.79509517902803	-1.12410258022099	-3.00126082461210
C	-1.12405540232819	-2.79502132230800	-3.00142011223734
C	1.12416119513660	-2.79501392176222	-3.00141865787570
C	0.70659208282623	-4.19266820348901	-3.13837680590149
C	-0.70647594752064	-4.19266203949079	-3.13848482459468
C	-0.70648103334124	4.19270621566492	-3.13809540423690
C	0.70658658510135	4.19270761813691	-3.13805115017705
C	1.12415531577314	2.79504348201195	-3.00120939092002
C	-1.12405458152307	2.79504131965980	-3.00125705762742
C	-1.43760334197940	-5.38263295578742	-3.25858764842273
C	-0.72283015490151	-6.59021389069069	-3.35591197926893
C	0.72296183822206	-6.59018511032569	-3.35616707974577
C	1.43772539141777	-5.38260341629848	-3.25878869905869
C	-5.38257644213456	1.43768486648542	-3.25850535992483
C	-6.59016610239707	0.72292540256597	-3.35582299497174
C	-6.59017478890737	-0.72286395171251	-3.35582775241983
C	-5.38259294623759	-1.43764126218300	-3.25854454661528
C	5.38272550892764	-1.43762809515114	-3.25827751464561
C	6.59028772158706	-0.72283826180713	-3.35571565395580
C	6.59024517223282	0.72294984975267	-3.35596879531360
C	5.38266209821274	1.43770201592489	-3.25853221433485
C	1.43772106967844	5.38266538991651	-3.25821126926622
C	0.72295494910356	6.59025704832373	-3.35546096641580
C	-0.72283220471165	6.59025672653812	-3.35551508872493
C	-1.43760673745769	5.38266678016408	-3.25829144005988
C	-2.67763522115134	-7.87085464096792	-3.81678713814039
C	2.67778817732898	-7.87095760944438	-3.81659894856492
C	7.87110291188173	-2.67769772583380	-3.81584869168682
C	7.87095009564237	2.67775189607151	-3.81666174604675
C	2.67773920803285	7.87100752215480	-3.81611668779336
C	-2.67759304780361	7.87103351602536	-3.81620013867687
C	-7.87092620748268	2.67776303664640	-3.81624943354117
C	-7.87094003021078	-2.67766046803824	-3.81641444425127
H	-2.53447805727431	-5.35108441396239	3.27549793856161
H	2.53439856322365	-5.35104801081005	3.27571136469208
H	-5.35106757178347	2.53439076556942	3.27567745308791
H	-5.35104874670090	-2.53447824349841	3.27567657284983
H	5.35093461671472	-2.53446762450266	3.27604729672195
H	5.35088163542651	2.53438206710617	3.27682527084616
H	2.53438756451692	5.35099966364352	3.27553130121957
H	-2.53447796689344	5.35099774898871	3.27553130559484
H	-2.88581466563194	-7.31486283686439	4.75579200913657
H	-3.32296966769204	-7.46179943780097	3.00801164400405
H	-2.91447619064432	-8.94052329554030	3.95917518853582
H	2.88565108707433	-7.31489488231044	4.75597469768976
H	3.32294426326425	-7.46173834585351	3.00822090989426
H	2.91439726500318	-8.94051513170448	3.95927792436515
H	8.94051029707127	-2.91459963500525	3.95857310083539
H	7.46159342879543	-3.32298195439150	3.00772490206474
H	7.31498443479414	-2.88598299718296	4.75555949049007
H	8.94041271480085	2.91434144364930	3.95974704271525
H	7.46167031646650	3.32305357702340	3.00877727798072
H	7.31474329364418	2.88548185955234	4.75644519239384
H	2.91436648861118	8.94059322965764	3.95843745110742
H	3.32304808227323	7.46160012648318	3.00784077503730
H	2.88547175983954	7.31513172194995	4.75554754540825
H	-2.91445380187578	8.94058468145779	3.95847544497701

H	-3.32314244551387	7.46160854504101	3.00785577209405
H	-2.88555699978334	7.31511014758825	4.75555852958778
H	-7.31498914421563	2.88560764155410	4.75568015817869
H	-7.46182188132457	3.32296847400792	3.00793988059691
H	-8.94059887528695	2.91434500062112	3.95895599363812
H	-7.31502311742867	-2.88576587914566	4.75560887585287
H	-7.46175690754027	-3.32303963112207	3.00783947181834
H	-8.94059138206646	-2.91448975137571	3.95880126104788
H	1.97473541490790	-5.56215890125267	0.00052625178803
H	5.56213895843994	-1.97470956534264	0.00003504967328
H	-5.56212994552250	-1.97468084188915	0.00012116032902
H	-1.97476353155911	-5.56216712105586	0.00004838375292
H	5.56215674173343	1.97473703905146	0.00027944624422
H	1.97467828375016	5.56211226084160	0.00017183211045
H	-1.97470158604172	5.56212435026660	0.00010554091598
H	-5.56213474268658	1.97470416323262	0.00018927141894
H	2.98263353564111	-7.44687672091211	0.90069951707894
H	2.98278114210074	-7.44677768653634	-0.90035421053791
H	4.20795617750242	-8.42685434121222	0.00021813397996
H	7.44667002008174	-2.98248597872795	0.90090297523781
H	7.44696613703195	-2.98278246173807	-0.90014288970233
H	8.42686744102263	-4.20786461804312	0.00074087006912
H	8.42682999670017	4.20793812986203	0.00049953261485
H	7.44691734366025	2.98269759578275	-0.90014687604829
H	7.44668363468014	2.98270569739568	0.90091498385455
H	4.20780513650847	8.42685689162891	0.00017119838986
H	2.98261663149264	7.44680702286812	-0.90039782333422
H	2.98256401481076	7.44679059787643	0.90065685041717
H	-4.20785915893508	8.42683857373982	0.00016749627122
H	-2.98263782172580	7.44683272554057	-0.90040552309537
H	-2.98262721421555	7.44675734513200	0.90064956759342
H	-8.42684026473463	4.20789122452744	0.00011379897871
H	-7.44681429164469	2.98259390817174	-0.90033455763555
H	-7.44679900984965	2.98271785752088	0.90072102043121
H	-7.44682096614228	-2.98260625884144	0.90058525149408
H	-7.44681976826908	-2.98258559644223	-0.90046317936652
H	-8.42687966732984	-4.20781955713288	0.00004705521852
H	-2.98273570098541	-7.44680567727340	0.90059479224991
H	-2.98276430638378	-7.44684655192603	-0.90045790733003
H	-4.20801034860629	-8.42683879377448	0.00010964009656
H	-2.53438497500011	-5.35104290898657	-3.27542735337691
H	2.53449270594020	-5.35093488463292	-3.27639143653861
H	-5.35091877150160	2.53445517397528	-3.27594791782455
H	-5.35095664117783	-2.53441263338618	-3.27597783195385
H	5.35108505115846	-2.53439581518290	-3.27596829579226
H	5.35094117944848	2.53445885610091	-3.27674051719096
H	2.53449387876867	5.35103534830790	-3.27556073956884
H	-2.53437799007096	5.35103584654537	-3.27575638214785
H	-3.32287154559770	-7.46179942140802	-3.00850036675506
H	-2.88570370908495	-7.31451699882834	-4.75625060357484
H	-2.91437042318369	-8.94033419533081	-3.95995619779273
H	3.32300401819619	-7.46169939496548	-3.00839955502959
H	2.88588822207740	-7.31486324813056	-4.75620037238041
H	2.91451907422099	-8.94047633294330	-3.95948309523872
H	8.94061678549610	-2.91442029628296	-3.95879929713771
H	7.31490161421193	-2.88588994964701	-4.75536068316168
H	7.46202234948665	-3.32286480965406	-3.00751880948947
H	8.94044469612594	2.91447738508184	-3.95975243943691
H	7.31467042783383	2.88564972762419	-4.75619260223066
H	7.46190140299345	3.32313129101218	-3.00848506919485
H	2.91446433153764	8.94051678082056	-3.95909738850620
H	2.88561859304797	7.31483375464384	-4.75571375167410
H	3.32313240228701	7.46186661640271	-3.00799837713878
H	-2.91430495384585	8.94054842517254	-3.95916061480824
H	-2.88544485107748	7.31488614047885	-4.75581920554508
H	-3.32301799197494	7.46187696093631	-3.00811515335999
H	-7.46160212193363	3.32301161347232	-3.00811224967779
H	-7.31488469055773	2.88579408480918	-4.75589689430262
H	-8.94044826567236	2.91451194150534	-3.95907982203571
H	-7.46168720500803	-3.32297234268049	-3.00829058847238
H	-7.31483644500121	-2.88563864052900	-4.75603671994987
H	-8.94045668616272	-2.91437975847819	-3.95933560554346
N	-1.99654292584872	-0.00004114282213	2.87250365621322
N	1.99646375373103	-0.00004232883955	2.87257782295736
N	-0.00004334096163	-1.99654957758419	2.87252739290041
N	-0.00003803211583	1.99646387309345	2.87254096115331
N	-2.39935807297652	-2.39935409077452	3.03309374998353
N	-2.39935563185658	2.39927628213862	3.03304877626443
N	2.39926726505911	2.39928001132534	3.03313758801274
N	2.39927156251082	-2.39934815478502	3.03320043444352
N	-1.37492895312808	-1.37495151710319	0.00003359555139
N	1.37493959411636	1.37490967650788	0.00011434999550
N	1.37493184846510	-1.37494997286988	0.00008498542187
N	-1.37492751794424	1.37491068621513	0.00006914091912
N	-0.00000034998577	-3.39499480166046	0.00008633775781
N	-3.39497180132886	-0.00001432131003	0.00007192134841
N	0.00000513671705	3.39495168624526	0.00012323049337
N	3.39497927796393	-0.00001833893592	0.00019117363874
N	-1.99645624352789	0.00000102902398	-2.87254079842668
N	1.99655678415191	-0.00000440378238	-2.87248995777867
N	0.00004893590652	-1.99650789518020	-2.87254167093737
N	0.00004987221195	1.99651193247582	-2.87247773522094
N	-2.39925816000848	-2.39930023399070	-3.03330336414471
N	-2.39925613956399	2.39931787556424	-3.03315463027928
N	2.39935274886652	2.39931964686486	-3.03310455816095
N	2.39936700675512	-2.39929740756585	-3.03324478946961
O	-1.29566603904968	-7.81035326871900	3.47910603118366
O	1.29560767374605	-7.81034296411791	3.47914289676050
O	7.81027376704673	-1.29570282673610	3.47898436806105
O	7.81023640860383	1.29561758559471	3.47943159473999
O	1.29561661606455	7.81033471107767	3.47840592756687
O	-1.29570872963540	7.81033197717938	3.47841521440049
O	-7.81037828361530	1.29559817382851	3.47878484339333
O	-7.81037308506092	-1.29570071357226	3.47876782413042
O	4.59212245729767	-6.43102278200245	0.00035906551446
O	6.43104123722074	-4.59207381903697	0.00047190246769
O	6.43100068296763	4.59211767150716	0.00025994688609
O	4.59203590037283	6.43103515993815	0.00017732745645
O	-4.59206526438838	6.43101294669230	0.00009900163649
O	-6.43101173482908	4.59207940704565	0.00009098818928
O	-6.43105807593207	-4.59204274202094	0.00004686005474
O	-4.59215356239365	-6.43100301098325	0.00007095254797
O	-1.29556581778915	-7.81025802567202	-3.47964604379793
O	1.29570818030117	-7.81026329587548	-3.47952139751910
O	7.81038783137311	-1.29558490867109	-3.47888808541237
O	7.81031488282590	1.29573294724898	-3.47929821710476
O	1.29572405825148	7.81033941053327	-3.47873441415200
O	-1.29559130975576	7.81034547931093	-3.47877095043166
O	-7.81024637680391	1.29569277976849	-3.47910628261064
O	-7.81025755716835	-1.29561451192284	-3.47917288179743
Y	-0.00002256067097	-0.00003246314088	1.68285369501251
Y	0.00003113520926	-0.00000882073099	-1.68281519035637

Table S10. BP86/def2-SVP geometry of ***g*-Y$_2$[(MeO)$_8$Pc]$_3$**.

C	-3.60744293632975	-2.26305974432049	3.17522424730590
C	-4.14990830812512	-0.95740959749504	3.14338827681462
C	-3.01818504355876	-0.03220598406284	3.07607116097097
C	-2.15253995025151	-2.10911124327839	3.09352491008598
C	2.14154492240307	2.11348709534417	3.09794921702676
C	3.59617106221398	2.26755516720427	3.18401954182850
C	4.13873271462155	0.96185014471998	3.15603648848911
C	3.00724056978851	0.03653260958700	3.08655697312491
C	0.02887172851346	-3.01053251016558	3.08366701128986
C	2.10578643791450	-2.14486353208050	3.10110311703609
C	2.25963455792550	-3.59957280876591	3.18620806840788
C	0.95400604850681	-4.14209875981719	3.15437044392255
C	-2.27095739765722	3.60402046288679	3.17328262917137
C	-0.96523731153763	4.14653591724510	3.14521821651439
C	-0.03984356253984	3.01489844362552	3.07907521880551
C	-2.11678893581042	2.14920707552992	3.09044670431600
C	0.74213289633329	-5.52854862604682	3.10698983504624
C	1.86730806534280	-6.36393889256641	3.08305594459555
C	3.19841878409343	-5.81476104628385	3.15181058160138
C	3.39409089445561	-4.42682500224453	3.20944580387428
C	-5.53625543669878	-0.74550711314272	3.09310417815998
C	-6.37158890738731	-1.87066246716420	3.06646757439634
C	-5.82256666794515	-3.20182353231916	3.13546849211593
C	-4.43475124489619	-3.39753145625342	3.19588072896320
C	5.52523589464981	0.74989794646812	3.11034384405636
C	6.36061083474182	1.87504311778277	3.08451650489268
C	5.81135917708264	3.20630580800593	3.14975579624437
C	4.42336760315142	3.40208465783614	3.20551593947496
C	-0.75325326122402	5.53294813479575	3.09689854968853
C	-1.87837621991934	6.36826520311046	3.06846138193063
C	-3.20968776230267	5.81914478104406	3.13378049667483
C	-3.40550303796993	4.43126701834683	3.19220897839422
C	0.53689771697418	-8.32467217999285	2.88151638519370
C	5.53833849602270	-6.28116185684185	3.15497265678446
C	8.32161647399069	0.54427528020331	2.88788697559906
C	6.27787793186631	5.54622596072381	3.14945836829984
C	-0.54745553999548	8.32893383767962	2.86952658137185
C	-5.54960619849302	6.28563859980108	3.13057295528530
C	-8.33195635784360	-0.54008870712446	2.86244663800473
C	-6.28900378682993	-5.54174007081431	3.13633279115898
C	-2.44867111211622	-3.44851459306094	0.01629296362171
C	-3.44772581978986	-2.44971503014241	-0.02266590315401
C	-2.75787989851278	-1.16989533169470	-0.01536478404105
C	-1.16904796143839	-2.75830226748058	0.01095207956397
C	1.16913293926075	2.75823061545832	0.01147941365688
C	2.44873118619887	3.44845023553731	0.02022575264638
C	3.44789962847890	2.44958721914474	-0.01407883290031
C	2.75802642433498	1.16978627692418	-0.00725557792083
C	1.16992036368073	-2.75800951291190	-0.00726162836484
C	2.75829551725042	-1.16912211652483	0.01694730670584
C	3.44851586396851	-2.44871721227585	0.02591490315899
C	2.44975630282510	-3.44785615160702	-0.01192078539075
C	-3.44843726511626	2.44869389432838	0.01066144301337
C	-2.44953040400994	3.44777582544347	-0.02491525523152
C	-1.16975368554970	2.75790059899588	-0.01536363868106
C	-2.75823598270947	1.16905805449925	0.00549261686836
C	2.77804808718953	-4.81117551126281	-0.05176290624969
C	4.13541241970153	-5.15278128664795	-0.03005992911561
C	5.15395899373187	-4.13375472524477	0.05043395418432
C	4.81187125769827	-2.77646315110456	0.06896825380401
C	-4.81097269328090	-2.77793787283695	-0.06557173168765
C	-5.15263761611994	-4.13531757403145	-0.04553344359856
C	-4.13379228517846	-5.15393011775067	0.03641650248736
C	-2.77653861339178	-4.81188679925260	0.05784816708503
C	4.81126311095130	2.77776002624628	-0.05320938410277
C	5.15288868036826	4.13515794108227	-0.03419683185110
C	4.13380171061048	5.15389860212830	0.04302125949444
C	2.77648190626600	4.81187938420127	0.06071756157200
C	-2.77761682830755	4.81107376379857	-0.06706016199972
C	-4.13502346068486	5.15276629397367	-0.04972793322620
C	-5.15384047467352	4.13386092299526	0.02884382408298
C	-4.81188177729022	2.77657386407475	0.04948522397986
C	3.67533951680076	-7.49367676499395	-0.09146734385287
C	7.49461741589391	-3.67287685631480	0.11554363639980
C	7.49384375624533	3.67493585251528	-0.09257610954966
C	3.67291646288044	7.49469248352171	0.10302512515491
C	-3.67476632923649	7.49361255031147	-0.11242088843417
C	-7.49465674529235	3.67307372875101	0.08769380725521
C	-7.49342157115818	-3.67523969648729	-0.11136311457017
C	-3.67304048374969	-7.49460277646098	0.10146569030777
C	-4.13910346893754	0.95608967632968	-3.15707467021656
C	-3.59693576177179	2.26191084463877	-3.18703397241790
C	-2.14225493090760	2.10840045591249	-3.10092091761669
C	-3.00731800254501	0.03121341669822	-3.08645516340287
C	3.01813572568409	-0.03562175392827	-3.07635224062813
C	4.15012250661053	-0.96057982712876	-3.14261210474892
C	3.60804707893948	-2.26644103365116	-3.17258269434634
C	2.15311284486136	-2.11282429480181	-3.09080224553395
C	-2.10519482801848	-2.14990385717862	-3.09790644287605
C	-0.02802728375368	-3.01491274839466	-3.07942370057915
C	-0.95282237303405	-4.14686045089159	-3.14859934012414
C	-2.25861338060475	-3.60477011947202	-3.18107624405634
C	0.96386583180856	4.14238116933698	-3.15082245626142
C	2.26976946579743	3.60026191082457	-3.17807649873109
C	2.11606792227124	2.14549898454678	-3.09357792926176
C	0.03884076010644	3.01052924986001	-3.08333344641057
C	-3.39280871277046	-4.43240886169890	-3.20337670369298
C	-3.19671836465943	-5.82021141011388	-3.14394052909208
C	-1.86544640154888	-6.36889439803731	-3.07437276320157
C	-0.74052892713958	-5.53318034906792	-3.09930426661993
C	-4.42448311198648	3.39615625835345	-3.21000117391587
C	-5.81240587594311	3.20003878815835	-3.15360970909329
C	-6.36124938782039	1.86870679186163	-3.08640811533488
C	-5.52552951642678	0.74378113264917	-3.11083490258889
C	4.43570128203793	-3.40068308421040	-3.19189962497560
C	5.82346869142437	-3.20446789049264	-3.13209086544572
C	6.37209668668274	-1.87305025785841	-3.06495140750945
C	5.53641670900755	-0.74818557821651	-3.09290142043595
C	3.40404192316341	4.42786211828102	-3.19767409819095
C	3.20774967447346	5.81573885200555	-3.14081497032953
C	1.87624591086596	6.36449693625506	-3.07640590620951
C	0.75140619196879	5.52877311010330	-3.10410191167949
C	-5.53649198840585	-6.28738534135869	-3.14820164827258
C	-0.53443423875275	-8.32891407665959	-2.86993479050231
C	6.29058341073983	-5.54424107974218	-3.12942472359742
C	8.33209034954302	-0.54164357593708	-2.86273383069361
C	5.54751168557326	6.28301528433659	-3.13771747309976
C	0.54464065882569	8.32488727785566	-2.87931788688089
C	-6.27962778871523	5.53982521001267	-3.15713407610283
C	-8.32176429575795	0.53756801041328	-2.88753814141668
H	-0.28334216772287	-5.91509909606704	3.05303667073715
H	4.39583232397732	-3.97944362021908	3.21512591259471
H	-5.92267480150377	0.28000301396903	3.03883780041707
H	-3.98741523128499	-4.39928826303825	3.20202056229132
H	5.91185688882302	-0.27568489370277	3.05901371180659
H	3.97596545790279	4.40382797286962	3.20864299295893
H	0.27237077918704	5.91945745167790	3.04551097333111
H	-4.40726037702670	3.98390324161970	3.19549383372619
H	-0.06681891447888	-8.12605477619758	3.79502057542117
H	-0.03469854655043	-7.96302738655577	1.99619525846438
H	0.70543939223670	-9.41351279139977	2.78730068196641
H	5.72606422085218	-5.65458559814133	4.05480924215948
H	5.80688293202785	-5.71366056927270	2.23918972616640
H	6.16862682424170	-7.18803832848772	3.20784525482475
H	9.41053200656278	0.71268854537870	2.79429275496924
H	7.96087423852508	-0.02938849316232	2.00352604942528
H	8.12221853205799	-0.05740581482948	3.80256868747044
H	7.18474591999290	6.17654233775681	3.20214167781743
H	5.71131227326550	5.81323883073856	2.23265566185729
H	5.65042994409507	5.73555406722501	4.04835365798377
H	-0.71579430471592	9.41768284612888	2.77388236370583
H	0.02703513940909	7.96666788994875	1.98632734681655
H	0.05336679008954	8.13112726570668	3.78511634131066
H	-6.17999427061049	7.19261710427658	3.18039653560232
H	-5.81561934822430	5.71686266447459	2.21484850690450
H	-5.73991235471519	5.66035619298163	4.03077753937836

H	-8.13500570182055	0.06279491410782	3.77686034064516
H	-7.96870453889316	0.03230930255802	1.97830319983006
H	-9.42062148136164	-0.70855435018333	2.76609069657613
H	-5.66464741750356	-5.73021195952950	4.03756198804963
H	-5.71925903285952	-5.80955383533971	2.22173415739636
H	-7.19601071509533	-6.17206256534087	3.18647606740286
H	1.98121506667491	-5.56369102642844	-0.07751158670036
H	5.56406300653881	-1.97931162383529	0.09451629683277
H	-5.56340001139285	-1.98105287683325	-0.09250546897141
H	-1.97946885497504	-5.56411448323696	0.08474515453863
H	5.56379029302699	1.98086841524106	-0.07672906803552
H	1.97935138044708	5.56416089578380	0.08409350512847
H	-1.98066206855183	5.56351301910307	-0.09146147590929
H	-5.56420968986680	1.97952575189502	0.07404788806234
H	3.10052898973764	-7.53622843347810	0.85696766542484
H	2.97734522570919	-7.41123023055588	-0.95449354165632
H	4.26868523033789	-8.42091129463879	-0.19210783567509
H	7.41032464544508	-2.97345006119164	0.97722714072827
H	7.53883132379302	-3.09965342644585	-0.83376684476648
H	8.42182952296085	-4.26578131584704	0.21894464515807
H	8.42115711371711	4.26805322122980	-0.19383117800486
H	7.41211073002263	2.97494577955644	-0.95405185159738
H	7.53561632136012	3.10233424885658	0.85722354784710
H	4.26571907235808	8.42207674476752	0.20546328565503
H	3.10097452204614	7.53711445056703	-0.84713998888557
H	2.97234616746833	7.41205729267567	0.96393997255643
H	-4.26782314571570	8.42072641008314	-0.21582566820038
H	-2.97425218705345	7.41021712790616	-0.97330936709745
H	-3.10272734728001	7.53723911835675	0.83762933626990
H	-8.42214504983176	4.26605209688941	0.18816771271251
H	-7.53623096068431	3.09926858488988	-0.86139046869453
H	-7.41274070652725	2.97419597142308	0.95005332973434
H	-7.53787979139722	-3.10096965273856	0.83730414429317
H	-7.40927101292216	-2.97674384281532	-0.97381605919740
H	-8.42044495478559	-4.26853971523689	-0.21418509518338
H	-2.97533417152098	-7.41064985364090	0.96457728059647
H	-3.09793172212194	-7.53841569928952	-0.84672354153620
H	-4.26614052229244	-8.42186266167479	0.20331078404053
H	-4.39469365414564	-3.98536145086784	-3.20972043762719
H	0.28506561041029	-5.91932273584945	-3.04468186528597
H	-3.97737376969281	4.39802514511538	-3.21438250819961
H	-5.91181277542408	-0.28184645003072	-3.05788613201291
H	3.98869262926198	-4.40259287887218	-3.19673560484286
H	5.92252710827028	0.27752232954464	-3.04021067841122
H	4.40594771292485	3.98082467704309	-3.20019395803941
H	-0.27436254376962	5.91498822603178	-3.05342091032781
H	-5.80573857075116	-5.71876984603301	-2.23331851196679
H	-5.72392906388105	-5.66205968529441	-4.04897062190860
H	-6.16645456102464	-7.19453582769340	-3.20023272410876
H	0.03693173250471	-7.96581948034450	-1.98506177434865
H	0.06931750320257	-8.13138117449194	-3.78365035044014
H	-0.70263240612023	-9.41767452405288	-2.77419927625090
H	7.19777156040276	-6.17437781527505	-3.17861134563955
H	5.66623752265010	-5.73432153135183	-4.03032678580230
H	5.72096985217258	-5.81077644180463	-2.21436891502321
H	9.42081109968258	-0.70968056111761	-2.76624445895239
H	8.13491157780596	0.06009200404237	-3.77785589283025
H	7.96876233771512	0.03171773011103	-1.97924268845576
H	6.17759584083969	7.19014374229096	-3.18865356660443
H	5.73806622382979	5.65665481484888	-4.03711594386867
H	5.81366841633340	5.71549583016725	-2.22125724688767
H	0.71260877609012	9.41377199361056	-2.78455933574289
H	-0.05608955972628	8.12614840847220	-3.79476798469296
H	-0.02975230883977	7.96315591874607	-1.99583536977686
H	-5.71299379012755	5.80858700206370	-2.24088431850997
H	-5.65238050322933	5.72779845058961	-4.05645168697486
H	-7.18669360815801	6.16977744283735	-3.21075236528130
H	-7.96058222675113	-0.03469670385357	-2.00245636563374
H	-8.12237540151979	-0.06533133284949	-3.80141801217830
H	-9.41071604332954	0.70573561112321	-2.79393072304176
N	-1.84626967897210	-0.76277704543803	2.98014841028192
N	1.83564831077388	0.76696918638837	2.98577223986336
N	0.75955116800564	-1.83885583276342	2.98579645856941
N	-0.77017249860352	1.84306821626102	2.98015532408101
N	-1.29982624230252	-3.13648090877586	3.11610890158052
N	-3.14420497170030	1.29645365838590	3.10959072380298
N	1.28873923803107	3.14088479546550	3.11601871161556
N	3.13314116511004	-1.29209058466777	3.12251497349021
N	-1.37612150304746	-1.37658591589130	-0.00167777537918
N	1.37623306054012	1.37650301631235	0.00157127570756
N	1.37659383303459	-1.37622255686743	0.00367041765456
N	-1.37649449735655	1.37613377494201	-0.00376415080646
N	0.00052028116305	-3.39208601835545	0.00223326034614
N	-3.39198645556181	-0.00050862859236	-0.00601110818283
N	-0.00039525048966	3.39198835873487	-0.00234728268343
N	3.39208492255350	0.00041480348221	0.00592617121664
N	-1.83593188310115	0.76213017331524	-2.98687870068702
N	1.84646277912281	-0.76643050871506	-2.97920547018085
N	-0.75904848194276	-1.84331980531801	-2.98309748682709
N	0.76954753235097	1.83906883437614	-2.98294806381795
N	-3.13282299452691	-1.29749266473735	-3.12044806619362
N	-1.28978375081600	3.13604685915083	-3.12042285400800
N	3.14374458275673	1.29303451110461	-3.11168064064064
N	1.30070458109902	-3.14048979487210	-3.11184785044562
O	1.81721037645711	-7.72506061072075	2.96029713613910
O	4.19363229760968	-6.74056231982744	3.11450015309149
O	7.72185279741570	1.82471206753106	2.96318802681290
O	6.73723657801392	4.20142097335472	3.11177033662910
O	-1.82795398246866	7.72928087123498	2.94466551646137
O	-4.20477314731812	6.74494542132720	3.09322776521534
O	-7.73247417590192	-1.82046779482365	2.94115248285123
O	-6.74830940404123	-4.19700137507928	3.09580664024516
O	4.61557421973147	-6.42622257648818	-0.08110775991330
O	6.42745250337927	-4.61343143271069	0.10475793186591
O	6.42637863628235	4.61518813634305	-0.08527332749105
O	4.61345191345180	6.42747910734210	0.09550480592111
O	-4.61501148989919	6.42615821426106	-0.10363098294218
O	-6.42746164148646	4.61361872388575	0.07923252481047
O	-6.42597256797859	-4.61545437564220	-0.09943580289391
O	-4.61358537225284	-6.42744305123746	0.08923030717245
O	-4.19165731079373	-6.74630524485376	-3.10637411875685
O	-1.81493516191535	-7.72982747722355	-2.94970793204591
O	6.74950899993789	-4.19931037469216	-3.09105715802978
O	7.73298118049711	-1.82228923614581	-2.93984386460190
O	4.20252255238351	6.74190960333148	-3.10102874192581
O	1.82534808360768	7.72561517137358	-2.95392652819392
O	-6.73857533817535	4.19494426945885	-3.11707186067489
O	-7.72245671805149	1.81810802334652	-2.96480799114129
Y	-0.00294594562809	0.00119133918453	1.74298612141875
Y	0.00300112892160	-0.00119750019603	-1.74302466013243

Table S11. BP86/def2-SVP geometry of ***g*-Y_2[(MeO)$_8$Pc]$_3^+$**.

C	-3.58984201430422	-2.29044092418638	3.16779630366956
C	-4.14191007688467	-0.98971270771584	3.14169157825650
C	-3.01650147634995	-0.05653268860412	3.06649993107939
C	-2.13442484805709	-2.12443900750367	3.08102892596446
C	2.12450628741335	2.12858459478776	3.08514022808060
C	3.57968209587042	2.29467859734414	3.17567865123845
C	4.13178529094208	0.99389488508706	3.15326429966334
C	3.00658617600162	0.06065007012898	3.07594831531282
C	0.05362835088109	-3.00951841486533	3.07292402012990
C	2.12148746867873	-2.12739330209202	3.08772849990951
C	2.28743399421941	-3.58264277725080	3.17725723263636
C	0.98674228919356	-4.13477853836050	3.15108115323114
C	-2.29767368930497	3.58689924262498	3.16610317465922
C	-0.99688579646184	4.13900176869297	3.14352492778537
C	-0.06353127406489	3.01367463921999	3.06932696063430
C	-2.13146770186838	2.13156245760032	3.07837828690582
C	0.78361940203435	-5.52227145506372	3.12300556791611
C	1.91583611046742	-6.35072844032425	3.10692292080950
C	3.24374565154589	-5.78992925320888	3.16174084511869
C	3.42864007718598	-4.39735989659842	3.20597097346950
C	-5.52934283068958	-0.78651198957383	3.11132158665784
C	-6.35779849143355	-1.91868110433780	3.09252845941589
C	-5.79713409564099	-3.24665491806167	3.14710762198474
C	-4.40466046888857	-3.43163807664275	3.19377038637841
C	5.51930878468805	0.79064087095456	3.12743321121124
C	6.34782512195339	1.92277648893885	3.10938859024268
C	5.78702253458268	3.25085073121868	3.16004839681520
C	4.39441591624169	3.43592331463725	3.20225477968565
C	-0.79364759295734	5.52648035998486	3.11521348192690
C	-1.92579893177127	6.35491182513460	3.09446716894545
C	-3.25389468630329	5.79417507465410	3.14523828097813
C	-3.43895374340741	4.40165624090703	3.19013006277444
C	0.60559601710808	-8.33517063978513	2.98781462076541
C	5.58818625207754	-6.24393661990837	3.17768867882545
C	8.33248349125020	0.61222488837977	2.99706739801900
C	6.24102380944664	5.59529746561251	3.16800993707916
C	-0.61517737677872	8.33935215682721	2.97958662320904
C	-5.59838337510082	6.24802143531769	3.14964789683605
C	-8.34189904385241	-0.60821160443649	2.97079281339738
C	-6.25100146971402	-5.59112573736801	3.15694143604551
C	-2.44806959259621	-3.44872125186566	0.01439809618929
C	-3.44840613174493	-2.44848742102552	-0.02120717606412
C	-2.75836232704569	-1.17011558277334	-0.01434441816788
C	-1.16975097446474	-2.75843280814470	0.01012332722327
C	1.16941984695805	2.75879937890121	0.01049512096949
C	2.44755829850351	3.44923407630746	0.01874672540295
C	3.44817029820317	2.44912267217720	-0.01227484795249
C	2.75824513070687	1.17060369971656	-0.00664954366936
C	1.17026581856882	-2.75811972772512	-0.00624416035556
C	2.75851446410043	-1.16935059404718	0.01576638108933
C	3.44885713010288	-2.44757675014358	0.02428212233259
C	2.44873414490265	-3.44808654176794	-0.00990992996544
C	-3.44906778039671	2.44806938240598	0.00880143168717
C	-2.44881869971720	3.44847738099930	-0.02390018522012
C	-1.17042926155501	2.75845689540716	-0.01471368328362
C	-2.75872322134153	1.16980924231232	0.00464180031185
C	2.77590201054988	-4.81179517151367	-0.04349758199752
C	4.13363089639762	-5.15386593538388	-0.02225048630606
C	5.15516892787553	-4.13183933468338	0.04417520804513
C	4.81259348565360	-2.77418627538934	0.06172666854613
C	-4.81202270461700	-2.77545359999530	-0.05975975599745
C	-5.15431612583686	-4.13314646296001	-0.04068028660764
C	-4.13265848300031	-5.15479575433595	0.02941520066502
C	-2.77498306915365	-4.81242825353131	0.05063370435397
C	4.81183469525784	2.77643274882417	-0.04649529586892
C	5.15373353999497	4.13426070744546	-0.02727709380651
C	4.13162706711176	5.15571054311843	0.03904137528520
C	2.77401584456291	4.81300220582106	0.05578032875460
C	-2.77580906892844	4.81211297996332	-0.06163550017719
C	-4.13357508097791	5.15430178666614	-0.04498592108841
C	-5.15528125990769	4.13251677138230	0.02226925722859
C	-4.81285199867107	2.77486238859907	0.04269744244570
C	3.68187020385483	-7.49901048002115	-0.07115519188720
C	7.50004535035740	-3.67913016318999	0.09685281611885
C	7.49899061944419	3.68258822401704	-0.06956282340251
C	3.67838792405454	7.50064104268926	0.08429550542102
C	-3.68129414057813	7.49938491337328	-0.09000156290161
C	-7.50035723804260	3.67986870706177	0.06456156296899
C	-7.49931283279359	-3.68084834202920	-0.09101745550322
C	-3.68034682734681	-7.49982617512616	0.07744572635791
C	-4.13210834117181	0.98811313800741	-3.15489145244199
C	-3.58041315054769	2.28904289488052	-3.17914977731451
C	-2.12519010836537	2.12354489422456	-3.08827076113146
C	-3.00661942705664	0.05532204618958	-3.07629446413431
C	3.01655786730019	-0.05998554005963	-3.06620221617225
C	4.14227349300704	-0.99292839182676	-3.13972725980279
C	3.59058728697334	-2.29384656913160	-3.16412868209369
C	2.13510343192777	-2.12813110086109	-3.07796439513581
C	-2.12084030843383	-2.13247001246023	-3.08487650874991
C	-0.05267072936015	-3.01384060392791	-3.06910819083028
C	-0.98536414081572	-4.13952080395101	-3.14580758838498
C	-2.28627378738296	-3.58792209658716	-3.17248436170731
C	0.99567474107591	4.13482217071128	-3.14948120612364
C	2.29657484745598	3.58297127372113	-3.17177014353047
C	2.13076631517527	2.12776053662131	-3.08172051013327
C	0.06258826289456	3.00935111805516	-3.07369011910447
C	-3.42714820116500	-4.40318452359154	-3.19909697555092
C	-3.24161478438220	-5.79563303572335	-3.15326142224983
C	-1.91342925769410	-6.35583255854711	-3.09909601575401
C	-0.78160822325840	-5.52689504847387	-3.11673775841047
C	-4.39551986538804	3.42998624266491	-3.20699704867497
C	-5.78806831792542	3.24451577163673	-3.16450111779912
C	-6.34844813428637	1.91632591962342	-3.11225513086596
C	-5.51956452449591	0.78443950338478	-3.12897497731403
C	4.40571906029647	-3.43484976324942	-3.18862559262491
C	5.79812872381615	-3.24942558969852	-3.14182043583890
C	6.35839735356928	-1.92123350686869	-3.08851766207784
C	5.52964267229248	-0.78930685684958	-3.10900641980823
C	3.43769011731110	4.39787560841307	-3.19765757859287
C	3.25241311671112	5.79040839753573	-3.15465190250045
C	1.92424598678001	6.35096584948844	-3.10368921640754
C	0.79221203551359	5.52230728634781	-3.12274350383573
C	-5.58587583869979	-6.25051572360184	-3.16477301269470
C	-0.60214164064358	-8.33948773992515	-2.97891708462534
C	6.25283852780805	-5.59376525536724	-3.15137229865417
C	8.34217170920178	-0.61012420212481	-2.96820439545711
C	5.59683849778836	6.24460794478256	-3.16137475039280
C	0.61342394530309	8.33532786936859	-2.98946038083739
C	-6.24272680198879	5.58882490504359	-3.17458182665224
C	-8.33261541072743	0.60517998783119	-2.99858807687852
H	-0.23812245957512	-5.91980203191545	3.08177661597532
H	4.42723176234824	-3.94330096144026	3.21330597172163
H	-5.92672638547000	0.23530079750496	3.07042724341752
H	-3.95069493635433	-4.43027176681032	3.20091723067508
H	5.91686821616571	-0.23122487270944	3.08966622821295
H	3.94039937053811	4.43455365393373	3.20662062291129
H	0.22823649384201	5.92395480293121	3.07700323307574
H	-4.43758522667836	3.94765365049598	3.19446412976773
H	0.02834525677932	-8.11620431666669	3.91280442590210
H	0.00345011638271	-8.01547157617602	2.10708217119502
H	0.79433557821466	-9.42238738638155	2.92576974124724
H	5.76749210091215	-5.60817211838524	4.07170223978047
H	5.85815327199425	-5.68824289687904	2.25546250481680
H	6.21675120312504	-7.15021180347913	3.24517047662011
H	9.41978309059706	0.80082754309106	2.93609419251301
H	8.01424377111864	0.00748496402010	2.11757194755339
H	8.11202633940432	0.03759097294131	3.92333144208465
H	7.14725048924514	6.22412016419820	3.23373724968752
H	5.68584133136486	5.86194199760144	2.24451368957059
H	5.60473208738728	5.77778021243347	4.06100670500749
H	-0.80369754529409	9.42653146786106	2.91625780820521
H	-0.00963369908994	8.01930783477635	2.10130130891933
H	-0.04142988826617	8.12072681309436	3.90683060240213
H	-6.22734683220059	7.15434582730277	3.21259664055449

H	-5.86380412636274	5.69082078151925	2.22699669111890
H	-5.78199239705689	5.61366456419412	4.04379425203563
H	-8.12472660119481	-0.03183165207996	3.89674552841500
H	-8.02032977204965	-0.00531499160181	2.09125399598687
H	-9.42900729752119	-0.79677930863385	2.90637937351832
H	-5.61733060889525	-5.77245609081077	4.05203656558040
H	-5.69309814837425	-5.85889474917827	2.23539079146033
H	-7.15737448996884	-6.21992558500557	3.22082425679948
H	1.98014588357510	-5.56556026563162	-0.06283776634459
H	5.56603093297909	-1.97811337035074	0.08105755409448
H	-5.56557932865709	-1.97959733410751	-0.08320729346259
H	-1.97910063060697	-5.56595207460810	0.07425149338539
H	5.56568792984983	1.98077788771272	-0.06705923657693
H	1.97789103538206	5.56634947246109	0.07682104304441
H	-1.97997484988122	5.56574684508069	-0.08343637380237
H	-5.56637611447356	1.97892581628123	0.06438116384576
H	3.11084244789093	-7.54171086245198	0.87919634598165
H	2.98329646329998	-7.42310208864933	-0.93351198531045
H	4.28340121080122	-8.42047946046866	-0.16956148660304
H	7.42221093854590	-2.97889775466749	0.95768736485575
H	7.54436425121999	-3.10993817945464	-0.85452770549013
H	8.42154496466552	-4.28012179535895	0.19822074462350
H	8.42070852104025	4.28406284769458	-0.16597759333218
H	7.42539010110518	2.98333491876620	-0.93157094814053
H	7.53911653262740	3.11228983097128	0.88134307246871
H	4.27916109799164	8.42257865413243	0.18296813518356
H	3.10930614706532	7.54197326118224	-0.86729484255190
H	2.97806922389928	7.42524001664225	0.94528262970246
H	-4.28243505568869	8.42111340918532	-0.18837633895466
H	-2.98108064438676	7.42441933611860	-0.95111738091698
H	-3.11205038764457	7.54070169087590	0.86149384391385
H	-8.42234827758529	4.28104824527704	0.16023441277368
H	-7.54011378943229	3.10862506486357	-0.88579347615239
H	-7.42661075876960	2.98150438302812	0.92728350164230
H	-7.54243367931874	-3.10971535810840	0.85925767283217
H	-7.42269941579921	-2.98235492238269	-0.95338054214876
H	-8.42087551823079	-4.28215534942682	-0.18993499477913
H	-2.98284402076287	-7.42422121667768	0.94069589947337
H	-3.10814022974792	-7.54178140208977	-0.87222920526242
H	-4.28173845675213	-8.42152572674344	0.17456455182044
H	-4.42595318944148	-3.94958718204723	-3.20576205195663
H	0.24035983711599	-5.92391194825531	-3.07623544510280
H	-3.94181883421353	4.42875359738303	-3.21239615099111
H	-5.91677087896672	-0.23750022211135	-3.08953935204964
H	3.95203999167046	-4.43361918987415	-3.19460987287553
H	5.92672181829989	0.23265791188358	-3.06894435864206
H	4.43639460285296	3.94402989376394	-3.20207288771158
H	-0.22971499414857	5.91967933652546	-3.08460392459476
H	-5.85442418482038	-5.69351893679052	-2.24291691730951
H	-5.76694304411466	-5.61615045808776	-4.05942510105167
H	-6.21424508420189	-7.15710650266346	-3.22980071654405
H	0.00045059640749	-8.01843770208554	-2.09898226444060
H	-0.02567380152953	-8.12125746160904	-3.90456753772481
H	-0.79026181050761	-9.42673190716959	-2.91550010758877
H	7.15946486795214	-6.22223786059244	-3.21486048787652
H	5.61951075088484	-5.77548787028553	-4.04663120108202
H	5.69478530841072	-5.86160144308770	-2.22995122996711
H	9.42931102658952	-0.79837293870582	-2.90331687014941
H	8.12501255428390	-0.03492240462846	-3.89488652231329
H	8.02043625760984	-0.00626314127221	-2.08938795361247
H	6.22560467722805	7.15093880364704	-3.22617741282064
H	5.77979396234424	5.60898596337030	-4.05475479398838

H	5.86313250236014	5.68881451706872	-2.23811973335002
H	0.80188876471907	9.42259212204537	-2.92732362802212
H	0.03890406072239	8.11586431608647	-3.91602520179818
H	0.00873291322720	8.01614280142929	-2.11027746054356
H	-5.68767004941778	5.85649707179049	-2.25129273730639
H	-5.60643814625566	5.77064055915203	-4.06771574939725
H	-7.14912356735839	6.21733181513420	-3.24097682402508
H	-8.01412932037958	0.00163892053174	-2.11836746519898
H	-8.11198563112466	0.02955883349056	-3.92419855496154
H	-9.41999736172209	0.79343427268214	-2.93796240493479
N	-1.83751354171408	-0.77585940619115	2.96713432450560
N	1.82791488702997	0.77986394245617	2.97215187991449
N	0.77300844556361	-1.83070559550562	2.97203576184419
N	-0.78264709271692	1.83479347173076	2.96713749074573
N	-1.27493836867075	-3.14575171363266	3.10496326931634
N	-3.15279623461623	1.27200995689813	3.09947088228673
N	1.26494135397597	3.14991835765504	3.10533352830380
N	3.14276430352912	-1.26785090812467	3.11095474039338
N	-1.37584284000393	-1.37594105177884	-0.00155454270169
N	1.37567080152732	1.37631495397376	0.00137377246920
N	1.37603879667863	-1.37556388604953	0.00344810485980
N	-1.37621570817725	1.37592539457156	-0.00369188976261
N	0.00033756145162	-3.39190284621035	0.00238505178709
N	-3.39216113956360	-0.00023341681412	-0.00591353108094
N	-0.00059347853811	3.39224004937595	-0.00257887560739
N	3.39200258142485	0.00071163575340	0.00561907719589
N	-1.82818026092002	0.77505436183444	-2.97348272088104
N	1.83776723615217	-0.77948791050237	-2.96601373975870
N	-0.77246203273588	-1.83516590004530	-2.96959934633274
N	0.78201307483352	1.83080049381709	-2.96998283381346
N	-3.14239008727848	-1.27328678255032	-3.10935846676809
N	-1.26594366582212	3.14513355569869	-3.10987516326578
N	3.15239413651251	1.26853807967622	-3.10131363020619
N	1.27593677106507	-3.14971491157861	-3.10072582865632
O	1.87827379566249	-7.70868543400219	3.00844380073608
O	4.24129869455656	-6.70491818001335	3.13309853572508
O	7.70591931391707	1.88492161598083	3.01298584685341
O	6.70204517494829	4.24827636920609	3.12850032244140
O	-1.88789759770024	7.71282966686124	2.99556101773970
O	-4.25133935929243	6.70907102216685	3.11089202018374
O	-7.71556869880999	-1.88096816580915	2.99152897187643
O	-6.71201020400223	-4.24417982253110	3.11439631895198
O	4.61466015110945	-6.42196321720468	-0.06558009463733
O	6.42334603158082	-4.61232390979788	0.09107022320704
O	6.42190763584168	4.61533829002798	-0.06764337588478
O	4.61183386092625	6.42413446486957	0.08191394895710
O	-4.61430730696374	6.42252546359183	-0.08768291373242
O	-6.42367072196838	4.61308562383837	0.06189162366253
O	-6.42249112880592	-4.61389740762860	-0.08477195589124
O	-4.61341439366557	-6.42303646634689	0.07140252597503
O	-4.23876167150913	-6.71096318266614	-3.12180162556985
O	-1.87516244892051	-7.71367984842567	-2.99935257641107
O	6.71334334885639	-4.24663916414244	-3.10892901555586
O	7.71614818879579	-1.88305050054864	-2.98741326206011
O	4.24974219978191	6.70550295116139	-3.12218308922421
O	1.88621337732488	7.70896544581347	-3.00596743338309
O	-6.70337404763072	4.24170809088065	-3.13382295829828
O	-7.70655111407756	1.87810847316566	-3.01606437520897
Y	-0.00273682023057	0.00126554023734	1.72939651195519
Y	0.00268504273743	-0.00111223810846	-1.72954043135653

Table S12. BP86/def2-SVP geometry of ***g*-Y$_2$[(MeO)$_8$Pc]$_3$$^{2+}$**.

C	-3.56750422018571	-2.32670458191115	3.15690162275630
C	-4.13142151561971	-1.03124934224620	3.13712945301336
C	-3.01584378577745	-0.08793771969421	3.05346553797831
C	-2.11253363656363	-2.14576152575021	3.06502649661235
C	2.10310104640010	2.14937081432531	3.06854968522292
C	3.55780058911380	2.33044651638627	3.16439547179509
C	4.12170401569190	1.03493932868932	3.14865953972204
C	3.00634617099992	0.09155038977363	3.06306709607326
C	0.08504041864219	-3.00930165641712	3.06066150272095
C	2.14281901771002	-2.10598352032161	3.07180475873534
C	2.32370760234787	-3.56071966478365	3.16717543203042
C	1.02828004761436	-4.12469333555218	3.14775980578879
C	-2.33345434567758	3.56465346901223	3.15356135828064
C	-1.03787543013148	4.12841757143931	3.13805325219536
C	-0.09446495559293	3.01283960818546	3.05607436816599
C	-2.15236648408186	2.10968616185635	3.06139954857245
C	0.83507349562700	-5.51334265847123	3.14293831245233
C	1.97436846302283	-6.33422132535432	3.13782481904013
C	3.29867464086150	-5.76075695196475	3.17598812854838
C	3.47212961532843	-4.36351504103949	3.20312096895908
C	-5.52004477904839	-0.83798367622873	3.12919042923762
C	-6.34093727437525	-1.97723919629018	3.12123403437533
C	-5.76759778983109	-3.30159449564219	3.15970299271894
C	-4.37043149560101	-3.47512267402327	3.19000978816028
C	5.51033662426422	0.84161159552616	3.14558256795506
C	6.33130488198793	1.98081836963007	3.13817471941384
C	5.75792216269701	3.30526986479875	3.17227272557436
C	4.36066283127069	3.47890847032090	3.19779040117292
C	-0.84436347868363	5.51700706089584	3.13201939379195
C	-1.98344041354086	6.33808445957986	3.12130580867938
C	-3.30800501294293	5.76492927865019	3.15503180878351
C	-3.48185791970441	4.36774704384986	3.18357635430145
C	0.68601341256576	-8.33991960673640	3.10134997418342
C	5.64760068504067	-6.20264039658629	3.19407830301016
C	8.33689073727663	0.69215394590475	3.10749835182279
C	6.20007096170344	5.65418417478949	3.18451598385368
C	-0.69443405858242	8.34339337584697	3.08811376435423
C	-5.65686854836864	6.20740870943969	3.16337478458131
C	-8.34649122925048	-0.68878043122488	3.08122682939849
C	-6.20959366806797	-5.65052225821373	3.17497218243794
C	-2.46904318682496	-3.43305029471991	0.00681486974848
C	-3.46578803361306	-2.42602710693274	-0.02449477071178
C	-2.76690379736825	-1.15400384033026	-0.01914852728705
C	-1.18671743879522	-2.74989189913662	0.00295675561723
C	1.18645253004952	2.74994411386856	0.00405355318307
C	2.46872895871527	3.43312994192290	0.01172253179703
C	3.46560993164500	2.42611020620258	-0.01557507622663
C	2.76676157700268	1.15403947031823	-0.01136320243222
C	1.15392267427993	-2.76682800515392	-0.01179301881221
C	2.74981571387895	-1.18657766355821	0.00870779515508
C	3.43296160761473	-2.46888767951359	0.01631813804148
C	2.42597132705139	-3.46570455845555	-0.01413250622914
C	-3.43322412125201	2.46885723504578	0.00239827496387
C	-2.42618571129099	3.46569038792851	-0.02554450731944
C	-1.15412495589877	2.76684025153986	-0.01861655227740
C	-2.75001659712810	1.18658123343450	-0.00157197307950
C	2.74805258369398	-4.83148187109782	-0.04631027095646
C	4.10500525111404	-5.17904956073832	-0.02947015207380
C	5.13327575777224	-4.15754306846154	0.02708521770560
C	4.79451743617696	-2.79749771990847	0.04860555905460
C	-4.83151315050823	-2.74802487207436	-0.05953986256499
C	-5.17913147290639	-4.10498842374503	-0.04492795875794
C	-4.15772593743274	-5.13335637815410	0.01182739710301
C	-2.79772099897415	-4.79465243415985	0.03650872828249
C	4.83142099365337	2.74818581417467	-0.04623742121504
C	5.17891684403108	4.10519490929054	-0.03219393859391
C	4.15727750888007	5.13356191736871	0.01994439412170
C	2.79723706917523	4.79477634903320	0.04071624078885
C	-2.74823050707091	4.83144308731293	-0.05918542546002
C	-4.10526641798240	5.17894198347431	-0.04725230193897
C	-5.13367944868157	4.15738616232486	0.00564413892172
C	-4.79490742460785	2.79739498779771	0.02937338356302
C	3.65654155238401	-7.52755640529150	-0.05233839753208
C	7.48390847978582	-3.71771498500887	0.07615230009563
C	7.52742192153147	3.65671205604012	-0.05093774546840
C	3.71715917437818	7.48426383133310	0.06193821863629
C	-3.65686014803047	7.52742208064878	-0.07006053966794
C	-7.48444764375666	3.71738442597561	0.04597251840833
C	-7.52758473237880	-3.65650252013376	-0.07277802024289
C	-3.71810578401865	-7.48405497187007	0.06000040011091
C	-4.12941231428714	1.00333438743256	-3.15299425673622
C	-3.57248987173221	2.30206067228600	-3.17238733253888
C	-2.11592823699065	2.13089803946802	-3.08068052215194
C	-3.00752448604377	0.06827035432966	-3.07117517209013
C	3.01709972319321	-0.07206335248537	-3.06126155278594
C	4.13925008053560	-1.00722350122013	-3.13806502147575
C	3.58242580252094	-2.30599544262349	-3.15741882148334
C	2.12555143272188	-2.13472500082135	-3.07064665997395
C	-2.12797758522579	-2.12262713389802	-3.07771364850670
C	-0.06537836195564	-3.01422499918566	-3.06416899137487
C	-1.00038381422450	-4.13629735746027	-3.14418548368567
C	-2.29908098138442	-3.57940658197898	-3.16615083582710
C	1.01017536551010	4.13240447762386	-3.14658183014375
C	2.30896388522316	3.57553443719196	-3.16339720862609
C	2.13760517461923	2.11885728210290	-3.07359895808141
C	0.07494377318240	3.01043748053258	-3.06812041640762
C	-3.44380026726685	-4.38518873775063	-3.19709508579390
C	-3.26527081695286	-5.78198220382070	-3.15980721654606
C	-1.93850148424725	-6.35028357044234	-3.11362160696756
C	-0.80261806223822	-5.52528391554570	-3.12773932079172
C	-4.37817967236089	3.44682185441346	-3.20394780211684
C	-5.77505772177440	3.26826705007478	-3.17014297848844
C	-6.34346313119128	1.94144137380680	-3.12687808582622
C	-5.51843634507350	0.80556818898409	-3.14023321556067
C	4.38829205414965	-3.45074432210062	-3.18479782644487
C	5.78504401968317	-3.27205106823700	-3.14677323397426
C	6.35324750902125	-1.94513949309406	-3.10347961263001
C	5.52821215640452	-0.80934661959005	-3.12104139812504
C	3.45375999567603	4.38130741943733	-3.19143742921783
C	3.27506257680771	5.77814455852900	-3.15665935956488
C	1.94811204505136	6.34645277754094	-3.11593712395031
C	0.81231754681654	5.52140489670741	-3.13283678780417
C	-5.61375073493996	-6.22789884831239	-3.19864270819569
C	-0.64448697567046	-8.35137738914378	-3.05618109916885
C	6.23110058090643	-5.62059138827241	-3.17949311949582
C	8.35417812825843	-0.65085403378374	-3.04686922272317
C	5.62364862350771	6.22409505573897	-3.18749214591284
C	0.65377721882282	8.34750919471819	-3.06525539255140
C	-6.22082361234428	5.61680319814025	-3.20669127387538
C	-8.34466548381318	0.64733724077881	-3.07553793525350
H	-0.18265499075290	-5.92215968880067	3.11668023464421
H	4.46761626721019	-3.90279365304923	3.21216844133785
H	-5.92872402528828	0.17979695924674	3.10277480556587
H	-3.90980456831235	-4.47064913921071	3.19937947256554
H	5.91911797749267	-0.17620778422127	3.12241324988443
H	3.90001324821209	4.47445243829325	3.20385017353117
H	0.17355205626637	5.92556733894801	3.10912135488518
H	-4.47748385660733	3.90727386932255	3.18954422303991
H	0.13160237403528	-8.09833038729374	4.03377445993038
H	0.05898096344767	-8.06211999130143	2.22442221322730
H	0.89534505492459	-9.42424555714942	3.07227839047385
H	5.82518723413617	-5.55654862651291	4.08009569277303
H	5.91189170689485	-5.65977438318363	2.26296972434027
H	6.27547411014568	-7.10788255054822	3.27259967300404
H	9.42127292797718	0.90126632003749	3.07896350950795
H	8.05986550165388	0.06286291999961	2.23194713782092
H	8.09428491931119	0.14023804507581	4.04113922587255
H	7.10533461105043	6.28215241593207	3.26203335637361
H	5.65790244167412	5.91609072778770	2.25234143202814
H	5.55338038195848	5.83417858972239	4.06961147433543
H	-0.90332362590887	9.42774470614450	3.05688556793448
H	-0.06392649500858	8.06433355841140	2.21407708568987
H	-0.14389339587204	8.10276107399793	4.02307762819529
H	-6.28481965857852	7.11294022592133	3.23783220704504

H	-5.91764178661600	5.66296350692233	2.23221674099818
H	-5.83809195746171	5.56293373107643	4.04983622347951
H	-8.10710294621230	-0.13524343891371	4.01473899745966
H	-8.06656958191715	-0.06094306687643	2.20554769337035
H	-9.43075486465725	-0.89803223855870	3.04937007586694
H	-5.56566366880200	-5.82884549476788	4.06241648598509
H	-5.66447755878534	-5.91409479955955	2.24497523031326
H	-7.11504528401712	-6.27842472244994	3.25079108778500
H	1.95147433046799	-5.58440551544038	-0.06423605192406
H	5.55121028947631	-2.00444404821988	0.06796928931609
H	-5.58439513459770	-1.95140276652727	-0.07713708729114
H	-2.00469864267191	-5.55138414775870	0.05552623417782
H	5.58441116257180	1.95160205368247	-0.06079390093698
H	2.00413101395466	5.55149742444306	0.05640109582209
H	-1.95163555684501	5.58441328175193	-0.07416532767846
H	-5.55162630228582	2.00430154752536	0.04580386034854
H	3.09625494543322	-7.56341874153410	0.90442023874191
H	2.95129388590769	-7.46190259196888	-0.90934903058358
H	4.26361852534014	-8.44510544243424	-0.14795912014843
H	7.41156221854096	-3.02991142423571	0.94636765129950
H	7.53206450527609	-3.13893887948296	-0.86873047244008
H	8.39747418125032	-4.33118588767892	0.16851160965824
H	8.44511053251984	4.26351273610616	-0.14697020921738
H	7.46290311002091	2.94913635380325	-0.90611935490502
H	7.56194874547448	3.09904004449424	0.90738647013392
H	4.33040486867682	8.39813195055002	0.15279746563796
H	3.13986001385704	7.52982603890382	-0.88397978434570
H	3.02800926365661	7.41420902267006	0.93128239540642
H	-4.26359332280892	8.44491113515780	-0.16840327780222
H	-2.94842214235401	7.46134778482668	-0.92441103145499
H	-3.10013068779555	7.56372072048821	0.88873944398332
H	-8.39838573438512	4.33083765418116	0.13469951293270
H	-7.52905603728715	3.13831481475363	-0.89891090816175
H	-7.41534996490913	3.02988662695770	0.91669459893480
H	-7.56564699349577	-3.09741680791599	0.88459237194944
H	-7.45996449687500	-2.95016305055211	-0.92873909560377
H	-8.44490804063250	-4.26345735637945	-0.17129112566886
H	-3.03183031136316	-7.41242161564029	0.93147760835872
H	-3.13769277923879	-7.53156071036650	-0.88390986229869
H	-4.33180129902733	-8.39764614201937	0.15059309109216
H	-4.44063570705192	-3.92744912731478	-3.20907952611632
H	0.21639408625284	-5.93066962002525	-3.09818021251061
H	-3.92038040768331	4.44365476414352	-3.21380238588151
H	-5.92394848990269	-0.21345818029253	-3.11298399020233
H	3.93064118516765	-4.44764345891041	-3.19471671308323
H	5.93352642626676	0.20976506642607	-3.09397533097783
H	4.45064873795912	3.92359303025983	-3.19941631544828
H	-0.20679662003628	5.92683890478687	-3.10770591487995
H	-5.89181864799058	-5.67234599873082	-2.27914900236209
H	-5.77982573503421	-5.59473288255844	-4.09596387184605
H	-6.23835762768234	-7.13570109719839	-3.27273125784748
H	-0.01771697554012	-8.06141274276891	-2.18290310316494
H	-0.09110028690947	-8.11871933285996	-3.99153912039940
H	-0.85000346152081	-9.43602119455852	-3.01444413079175
H	7.13898727161966	-6.24533534672185	-3.25134934343505
H	5.59863046799066	-5.78892000342148	-4.07688680331097
H	5.67484699676181	-5.89641022447961	-2.25974024433111
H	9.43878768388500	-0.85617311806401	-3.00329965789839
H	8.12260963567138	-0.09976163805995	-3.98385088257062
H	8.06309699242998	-0.02199878613894	-2.17545746266721
H	6.24848052932904	7.13182239586432	-3.26059166685023
H	5.79296684540620	5.58970730865360	-4.08334549029072

H	5.89843894670119	5.66981041852149	-2.26624189362182
H	0.85905184606732	9.43221572991393	-3.02395704464407
H	0.10357810504929	8.11381308527166	-4.00223437513151
H	0.02410121629136	8.05839511936746	-2.19378226580451
H	-5.66745656287633	5.89362494660936	-2.28549519451829
H	-5.58551716928203	5.78405936019426	-4.10227928605408
H	-7.12843283044875	6.24153217403731	-3.28210660649024
H	-8.05667845573126	0.01964915599116	-2.20226359770184
H	-8.10986032456520	0.09494986098923	-4.01095080841549
H	-9.42940585375429	0.85279045150701	-3.03606684235108
N	-1.82774819664953	-0.79529629369947	2.95019781622378
N	1.81859799873533	0.79875379053187	2.95499952227760
N	0.79246292710729	-1.82145800347373	2.95514303674090
N	-0.80162020004047	1.82487072706874	2.95005024268804
N	-1.24202546027389	-3.15884138055444	3.09090721694828
N	-3.16548755060638	1.23912945507074	3.08417203803432
N	1.23250070539151	3.16244595431982	3.09024837950245
N	3.15586532530879	-1.23542832788478	3.09654091257255
N	-1.38567022438210	-1.36542161408395	-0.00939335479010
N	1.38550041390406	1.36546753564048	-0.00626729425631
N	1.36536070379265	-1.38557121947159	-0.00464992238506
N	-1.36553069086562	1.38559162221752	-0.01091448109335
N	-0.02324996104539	-3.39189618463520	-0.00338678382413
N	-3.39200087975848	0.02313485268112	-0.01060918855504
N	0.02300386414023	3.39192927920270	-0.00677043694475
N	3.39182263006067	-0.02311457549574	0.00044041457666
N	-1.82437407003752	0.78060184759619	-2.96689742867947
N	1.83361400697794	-0.78426892770433	-2.95980933161861
N	-0.77777403109796	-1.83080681534333	-2.96340560610059
N	0.78705155209118	1.82718881978718	-2.96331871985685
N	-3.14661694034002	-1.26231588751936	-3.10232559572071
N	-1.25556141558358	3.14955205287644	-3.10236072865933
N	3.15630079953768	1.25848562292574	-3.09377970923316
N	1.26524288285573	-3.15341746696234	-3.09366788009344
O	1.94963193911232	-7.68898482494085	3.06739317525959
O	4.29866366866962	-6.66530420756708	3.15515512037719
O	7.68615567031208	1.95576098560501	3.06952303626321
O	6.66259595722502	4.30510020594862	3.14946320287386
O	-1.95809537719966	7.69278053402890	3.04978546701881
O	-4.30768652421226	6.66967874833396	3.12891408272688
O	-7.69553180462815	-1.95239662151962	3.04766766016154
O	-6.67212462972986	-4.30153930093331	3.13601110952580
O	4.58391644743263	-6.44280852985188	-0.06418981723391
O	6.39451767268384	-4.64085849678568	0.05973824379929
O	6.44270123627616	4.58405944074047	-0.06674506993846
O	4.64043291106968	6.39492598136110	0.04987676776159
O	-4.58413929946499	6.44263858753060	-0.08478580199847
O	-6.39507354857339	4.64060131217464	0.03331675685697
O	-6.44280399891381	-4.58385256809879	-0.08330487581008
O	-4.64115345188946	-6.39460063155839	0.04276562348015
O	-4.26368506708263	-6.68750087081155	-3.13618327160831
O	-1.91004074469652	-7.70434215920302	-3.02923227891342
O	6.69059473434329	-4.27035297198290	-3.12003449340648
O	7.70721284887011	-1.91639642793361	-3.01763290399150
O	4.27334739834799	6.68372498291786	-3.13053088426554
O	1.91929470083041	7.70062028852439	-3.03332396821831
O	-6.68060342986468	4.26666285129965	-3.14715188799544
O	-7.69772175625739	1.91287749339255	-3.04575028403137
Y	-0.00258878516059	0.00093161144383	1.71704850037764
Y	0.00259307326562	-0.00093557933189	-1.72518546187440
